# Molecular Mechanisms of Tungsten Toxicity Differ for *Glycine max* Depending on Nitrogen Regime

**DOI:** 10.3389/fpls.2019.00367

**Published:** 2019-04-02

**Authors:** Julian Preiner, Stefanie Wienkoop, Wolfram Weckwerth, Eva Oburger

**Affiliations:** ^1^Division of Molecular Systems Biology, Department of Ecogenomics and Systems Biology, University of Vienna, Vienna, Austria; ^2^Department of Forest and Soil Sciences, Institute of Soil Research, University of Natural Resources and Life Sciences Vienna, Tulln, Austria; ^3^Division of Terrestrial Ecosystem Research, Department of Microbiology and Ecosystem Science, University of Vienna, Vienna, Austria

**Keywords:** glycine max, tungsten toxicity, N-assimilation, nitrate reductase, ^15^N natural abundance, symbiotic N_2_ fixation, starch accumulation

## Abstract

Tungsten (W) finds increasing application in military, aviation and household appliance industry, opening new paths into the environment. Since W shares certain chemical properties with the essential plant micronutrient molybdenum (Mo), it is proposed to inhibit enzymatic activity of molybdoenzymes [e.g., nitrate reductase (NR)] by replacing the Mo-ion bound to the co-factor. Recent studies suggest that W, much like other heavy metals, also exerts toxicity on its own. To create a comprehensive picture of tungsten stress, this study investigated the effects of W on growth and metabolism of soybean (*Glycine max*), depending on plant nitrogen regime [nitrate fed (N fed) vs. symbiotic N_2_ fixation (N fix)] by combining plant physiological data (biomass production, starch and nutrient content, N_2_ fixation, nitrate reductase activity) with root and nodule proteome data. Irrespective of N regime, NR activity and total N decreased with increasing W concentrations. Nodulation and therefore also N_2_ fixation strongly declined at high W concentrations, particularly in N fix plants. However, N_2_ fixation rate (g N fixed g^−1^ nodule dwt) remained unaffected by increasing W concentrations. Proteomic analysis revealed a strong decline in leghemoglobin and nitrogenase precursor levels (NifD), as well as an increase in abundance of proteins involved in secondary metabolism in N fix nodules. Taken together this indicates that, in contrast to the reported direct inhibition of NR, N_2_ fixation appears to be indirectly inhibited by a decrease in nitrogenase synthesis due to W induced changes in nodule oxygen levels of N fix plants. Besides N metabolism, plants exhibited a strong reduction of shoot (both N regimes) and root (N fed only) biomass, an imbalance in nutrient levels and a failure of carbon metabolic pathways accompanied by an accumulation of starch at high tungsten concentrations, independent of N-regime. Proteomic data (available via ProteomeXchange with identifier PXD010877) demonstrated that the response to high W concentrations was independent of nodule functionality and dominated by several peroxidases and other general stress related proteins. Based on an evaluation of several W responsive proteotypic peptides, we identified a set of protein markers of W stress and possible targets for improved stress tolerance.

## Introduction

Increasing environmental contamination with heavy metals and other xenobiotics has become a major concern for agricultural production and human food safety. Thus, it becomes a necessity to understand how crop plants cope with environmental pollutants. Despite the myriad of applications tungsten finds in different industries, ranging from everyday household appliances to high tech and military products, the transition metal W has received only little attention, and information about the behavior of tungsten in the plant-soil environment is limited. Besides discharge of W utilizing industries and W mining sites, tungsten finds entry into the environment due to disposal of W-containing products, military activities, coal driven power plants and soil fertilizer application (Koutsospyros et al., 2006; Strigul et al., 2010). Background concentrations of tungsten in soil range from 0.1 to 2.7 mg kg^−1^; however, in war zones, firing ranges, mining sites and agricultural soils concentrations have been found to be exceeding these background levels considerably (10–2,000-fold) (Kennedy et al., 2012). Tungsten concentrations in soils from abandoned mining sites in Portugal are reported to range from 0.8 to 684 mg kg^−1^(Pratas et al., 2005). Typical ranges of W in agricultural soils in the European Union lie between 0.5 and 83 mg kg^−1^ due to fertilization with contaminated phosphate fertilizers (Senesi et al., 1988). Once in the environment, tungsten is mostly present as oxyanion in form of tungstate WO_4_^(−2)^ and its protolysis species as well as in form of various iso-or heteropolytungstate species. Tungstate is thus thought, at least in its unpolymerized form, to be readily taken up by plants similarly to other anions (Schwarz et al., 2007; Bevers et al., 2009).

Tungsten shares certain chemical properties regarding structure, electro negativity, ionic and atomic radii as well as range of oxidation states (−2 to +6) with the essential plant micronutrient molybdenum (Leffler and Kazantzis, 2015; Tallkvist and Oskarsson, 2015). Due to these similarities it is proposed and under *in-vitro* conditions already tested that tungsten is able to substitute the Mo-ion bound to the co-factor of important enzymes of the nitrogen, sulfur and carbon metabolism (McMaster and Enemark, 1998; Ataya et al., 2003; Siemann et al., 2003; Bevers et al., 2009). Similar substitutions of so called physiological ions were already observed for other non-physiologic heavy metals (e.g., replacement of calcium by strontium during heat stress, substitution of iron by uranium in ferritin, as well as replacement of magnesium by zinc, cobalt and nickel in RuBisco) (DalCorso et al., 2013; Cvjetko et al., 2014; Viehweger, 2014).

Leguminous plants such as soybean rely on six different molybdoenzymes: xanthine dehydrogenase, sulfite oxidase, nitrate reductase (NR), aldehyde oxidase and the mitochondrial amidoxime reducing component as well as the symbiotic nitrogenase (Bittner, 2014). While the first five enzymes share similar pterin cofactors (Mo-MPT) to which the Mo-ion is bound, the latter utilizes a Fe-Mo cofactor for nitrogen fixation (McMaster and Enemark, 1998; Hille, 2002). It was already shown that the N_2_ fixation activity of N_2_ fixing bacteria is reduced or inhibited by the presence of tungsten (Siemann et al., 2003; Ringelberg et al., 2009; Strigul et al., 2009), although some exchange experiments indicate that bacterial Mo-nitrogenase can be functional when Mo is substituted by W (Kletzin and Adams, 1996; Schwarz et al., 2007; Bevers et al., 2009). In symbiotic bacteria *B. japonicum* Harper and Nicholas (1978) even found an increase of acetylene reduction in soybean grown on 0.4 mM W. Regarding plant endogenous molybdoenzymes, previous work suggests that the replacement of molybdenum in Mo-MPT with tungsten causes an inhibition of the enzyme's catalytic function but not its production and thus renders it functionless (Harper and Nicholas, 1978; Deng et al., 1989; Zimmer and Mendel, 1999; Schwarz et al., 2007; Bevers et al., 2009; Xiong et al., 2012). As comprehensively reviewed by Xiong et al. (2012), this substitution is likely not only affecting nitrate reduction and nitrite oxide levels but, similar to other heavy metals, results in oxidative stress via increased ROS production by inhibition of ABA biosynthesis, purine metabolism and sulfur metabolism; however a comprehensive assessment of W induced metabolic changes has not been conducted so far. Literature suggests that W toxicity is depending on organism and dose applied. Considering their size, relatively quick growth and a high demand for Mo, leguminous plants appear to be particularly interesting for research into W uptake and toxicity regarding future efforts to safely and efficiently remove tungsten from contaminated sites (e.g., remediation of war zones, and waste disposal sites) (Strigul et al., 2009).

To date, most plant-related studies either focus on the effects of W substitution on nitrate assimilation (Harper and Nicholas, 1978; Deng et al., 1989) and ABA biosynthesis (Jiang and Heilmeier, 2007), or investigate the growth retarding effects of W as well as its general toxicity for higher organisms (Strigul et al., 2005; Bamford et al., 2011). While the negative effects of W on molybdoenzymes in plants an prokaryotes has received considerable attention, comprehensive information of W uptake, effects on plant metabolism and the mechanism of W toxicity on a molecular and proteome wide level is still missing. Additionally, information on the toxicity of tungsten for higher life forms and ecosystems in general, as well as our understanding of its effects on plant nitrogen cycle, plant-microbe interaction, metabolic processes apart of NR inhibition is limited.

In order to identify the effect of tungsten stress, we investigated soybean grown in semi—hydroponics with a major focus on root and nodules. In addition to the assessment of biomass production, starch and nutrient content, N_2_ fixation and NR activity, a proteomic analysis of roots and nodules was carried out. In particular, we were interested whether or not a functional symbiotic association with N_2_ fixing rhizobia influences resistance to W stress compared to nitrate fed plants with strongly reduced symbiotic interaction. Vice versa, we wanted to unravel, whether high W impacts both N regimes and the varying N-assimilation strategies differently.

## Materials and Methods

### Plant Culture and Sampling

Soybean (*Glycine max* cv. Primus obtained from Die SAAT Austria) seeds were soaked in diluted industrial Radicin inoculant (*B. japonicum*) (7.5 ml filled up to 100 ml with HQ water) for 5 h. For germination, seeds were sown into acid-washed (2% HNO_3_) plastic boxes containing perlite and a germination solution (0.6 mM CaSO_4_^*^ 2H_2_O, 0.002 mM H_3_BO_3_, pH 6.2, conductivity of 136.2 μS cm-1).

After 1 week seedlings were transferred into pots containing a substrate mixture of vermiculite and perlite in a 5/3 (v/v) ratio, and watered with a modified half strength Hoagland nutrient solution (N-free, pH 7.2, conductivity of 1066–1160 μS cm^−1^ between control and 0.5 mM W) (Hoagland and Arnon, 1950). After 2 weeks, seedlings were again inoculated by directly applying 1 ml diluted (as described above) industrial Radicin inoculant (*B. japonicum*) to each root.

To test the effects of different N regimes (N fertilization, N_2_ fixation) on tungsten uptake by the plants, three different W treatments (control, 0.1 mM, 0.5 mM) were applied for 6 weeks. One half of the plants (N fed) was supplied with 10 mM KNO_3_ from week two onwards, the other half (N fix) received 0.25 mM KNO_3_ for 2 weeks and then were only watered with N-free nutrient solution. For each treatment 5 biological replicates where prepared. The plants were grown under controlled conditions with a 12/12 light/dark cycle at 29/21°C and an approximate photosynthetic radiation of 400 μmol m^−1^s^−1^ at canopy level.

Tungsten concentrations were chosen based on a germination test (Data not shown) as well as literature data. The highest tungsten concentration (0.5 mM corresponding to 91.9 mg W L^−1^) was chosen because it was the highest concentration where plants still germinated and growth was still was not yet significantly hampered. The second tungsten concentration (0.1 mM W or 18.4 mg W L^−1^) was chosen as intermediate between the decided on highest concentration and the control.

### Sampling and Acid Digestion

Plants were harvested after 49 days as they reached the full pod stage (Fehr and Caviness, 1977). A subsample of roots, nodules and leaves was cut off using a stainless steel razorblade, transferred into a precooled 2 ml Eppendorf tube and immediately put on liquid nitrogen (LN_2_) for proteomic analysis and stored at −80°C.

The rest of the biomass was harvested for acid digestion and ICP-MS/OES analysis. Roots and nodules were washed and ultra-sonicated with CaCl_2_ (0.01 M) for 5 min followed by further 3 min of ultra-sonication with HQ-water. Roots, shoots and nodules were separated and dried at 60°C for 1 week and subsequently ground to a homogenous fine powder using a Retsch mill (Retsch MM20).

### Biomass Production and Growth

To evaluate the effect tungsten and nitrogen regimes had on biomass production, four parameters were measured: fresh and dry weight of root and shoot as well as length of the major root and shoot. Accordingly, also the nodule biomass production was assessed by counting and weighing the nodules.

### Strach Extraction

Aliquots of the dried plant material were used for determination of total starch content according to Nagler et al. (2015). Briefly, soluble sugars were removed by boiling approximately 15 mg of the plant material twice for 30 min with 80% ethanol at 80°C. The pellets were then re-suspended in 0.5 M NaOH and heated to 950°C for 30 min and subsequently acidified with 1 M CH_3_COOH and digested for 2 h at 55°C with amyloglucosidase. Starch content was then assessed by determination of glucose content by further incubating 200 μl of the obtained supernatant at 30°C with 400 μl glucose oxidase reagent (50 ml Tris-Glycerin-Buffer pH 7, 15 mg glucose oxidase, 1.5 mg peroxidase, 5 mg o-Dianisidin-HCl). After 1 h, the reaction was stopped by adding 800 μl of ice cold HCl and absorbance was determined at 540 nm against a calibration curve using a glucose standard processed in the same manner as the amyloglucosidase digests.

### Acid Digestion and ICP-Measurement

Approximately 200 mg of dry, ground plant material was used for acid digestion. The digestion matrix contained 5 ml HNO_3_, 1 ml H_2_O_2_ and one drop 1-Octanol. For biomass < 100 mg half of acid and peroxide was used. The digestions were performed using an open digestion unit (Velp Scientifica). Digests were filled up to a volume of 50 ml/25 ml with HQ-water resulting in ~6.5% HNO_3_. If dry weight was lower than 50 mg, digestion was performed with a Multiwave 3000 (64 MG5 rotor) using only 0.5 ml HNO_3_ (sub-boiled) and 0.1 ml H_2_O_2_. After the digestion, micro-digests were acidified with hydrofluoric acid to a final concentration of 0.01% in order to prevent polymerization or precipitation of W.

For measurements of nutrient and W content in nodules, biomass was not always sufficient to perform digestion for individual replicates. If present, nodules of treatments with low biomass (plants supplied with KNO_3_ and those treated with W) were pooled. Results only represent estimates and are thus not extensively discussed (Table S2). Micronutrients and W concentrations in the digests were measured with inductively coupled plasma mass spectrometry (ICP-MS; Perkin Elmer, Elan DRCe 9000, Waltham, MA, USA); macronutrients were analyzed by ICP-OES Optima 3000 XL (Perkin Elmer).

### Cytosolic Nitrate Reductase Assay

Nitrate reductase assay was performed using fresh plant material according to Sanderson and Cocking (1964) and Stöhr and Ullrich (1997). Briefly, 6 h into the light period a composed sample was taken from the three uppermost fully expanded trifoliates, taking one leaf each. Samples were ground and homogenized in a prechilled (−20°C) porcelain mortar at with an extraction buffer (1 ml/100 mg FW), and centrifuged at 4°C with 5,000 g for 5 min. The extraction buffer was used to stabilize the enzyme in solution and contained following substances in given concentrations HEPES 100 mM, EDTA 1 mM, sucrose 330 mM, riboflavin 10 μM, Na_2_MoO_4_ 1 μM, casein 1 g l-1, glycerin 10% (v/v), cysteine 10 mM, DTT 1 mM, Na-ascorbate 100 mM, PVPP 1.5% (w/v), PVP 5% (w/v), PMSF 1 mM. PH of the buffer was adjusted to 7.5 with NaOH and buffer prechilled at −20°C until usage. Protein content was determined as previously described using Bradford assay.

The NR-assay was performed using a reaction buffer containing 200 μl 0.1 M phosphate buffer, pH 7.5, 50 μl of 0.01 M NADH, 50 μl of 0.1 M KNO_3_ as well as 200 μl of enzyme extract making up a full volume of 500 μl (Sanderson and Cocking, 1964). A blind control for each sample was processed without adding NADH to the buffer. Samples and blinds were incubated for 30 min at 30°C.

The incubation was immediately stopped by adding 1 ml of a mixture (ratio 1:1) of 2.5% (w/v) sulfanilamide in 3.75 N HCl and 0.5% (w/v) N-(1-Naphtyl) ethylenediamin-dihydrochlorid (Snell and Snell, 1957). After another incubation of 10 min at room temperature the samples were centrifuged for 5 min with 10,000 g and the extinction determined at 540 nm. Absorption values where then converted into nmol of nitrite (NaNO_2_) by using a standard curve and the previously determined protein content.

### ^15^N Natural Abundance

Symbiotic N_2_ fixation and nodule fixation activity was estimated based on natural N isotope fractionation between plant organs using the ^15^N natural abundance method (Högberg, 1997). Briefly, dried and finely ground plant tissues were weighed into tin capsules and analyzed for N content and ^15^N:^14^N ratios by an elemental analyzer (EA 1110; CE Instruments, Milan, Italy) coupled via a ConFlo II interface (Finnigan MAT, Bremen, Germany) to a gas isotope ratio mass spectrometer (IRMS, DeltaPLUS; Finnigan MAT). Reference N_2_ gas was calibrated to the atmospheric N_2_ standard using certified IAEA reference materials. The relative abundance of ^15^N in plant samples was expressed in δ units, which denote the deviation in %0 of the sample ^15^N:^14^N ratio from that in atmospheric N_2_. The standard deviation of repeated measurements of a laboratory standard was below 0.15%0. The δ^15^N of total plants (roots + shoots + nodules) were calculated as the N mass-weighted mean of the δ^15^N of the respective plant parts (Högberg, 1997). The δ^15^N signatures were used to estimate the percentage of plant N derived from the atmosphere (%NdfA) as a proxy of N_2_ fixation/nitrogenase activity (Equation 1).

(1) $$\begin{array}{r} \%NdfA=\frac{\delta^{15}N_{ref}-\;\delta^{15}N_{fix}}{\delta^{15}N_{ref}-B}\;\times 100 \end{array}$$

Where δ^15^N_*ref*_ represents the δ^15^N value (−4.49) of non-nodulating (*Glycine max* cv Primus) grown under the same control conditions (including 20 mM NO_3_ supply) as the soy plants from the control and W treatments from the experiments as described above; δ^15^N_*fix*_ represents the δ^15^N value of the individual, experimental test plants (*Glycine max* cv Primus) and *B* is the δ^15^N value (−0.56) of soy grown with N_2_ as the sole source of N.

Nodule fixation activity (g N fixed g^−1^ nodule dwt) was calculated according to Equation (2)

(2) $$\begin{array}{r} Nodule\;fixation\;activity\;=\frac{\%NdfA{\;}^{*}\;N_{plant\;tot\;}}{{100{\;}^{*}M}_{nodule}} \end{array}$$

with N_*planttot*_ being the total amount of N accumulated during the growth period (mg N) and M_*nodule*_ being the dry nodule biomass (mg).

#### Extraction of Proteins

Frozen plant material was ground to a fine powder on LN_2_ using a porcelain mortar. Sixty milligrams (fresh-weight) were then used for protein extraction according to an adapted version of the integrative extraction procedure for proteins and metabolites as described by Morgenthal et al. (2007). All samples were extracted by adding 1 ml of extraction buffer containing methanol/chloroform/water (MCW) (2.5:1:0.5 [v/v/v]). After vortexing and centrifugation (14,000 × g, 4 min, 4°C), the supernatant was removed and the protein pellet washed three times with methanol/chloroform (1:1 [v/v]) and air-dried on ice.

The pellet from the MCW extraction was solubilized in 1 ml of protein extraction buffer (0.05 M TRIS/HCl, pH 7.6, 1.5% SDS, 1% ß-mercaptoethanol; 0.6 M sucrose) and incubated at room temperature for 15 min. After adding 1 ml of TE buffer equilibrated phenol (pH 7.5–8), the samples were incubated for 1 h at 37°C and later centrifuged (16,000 × g, 20 min, 30°C). The upper phenolic phase containing soluble proteins was covered with 6 volumes of ice-cold ammonium acetate in methanol and proteins precipitated at −20°C overnight. Following a 10 min centrifugation at 4°C with 4,000 g, the pellet was subjected to consecutive washing steps with 1 ml ice cold ammonium acetate, 1 ml ice-cold methanol and 1 ml acetone and then air dried at room temperature for 5–10 min. The pellet was re-dissolved in buffer containing 8 M urea and 100 mM ammonium bicarbonate (AmBic) for protein content determination, using a Quick Start Bradford protein assay from Bio-Rad Laboratories, Inc. and a Perkin Elmer photometer (EnSpire 2300 Multilable Reader). Protein content was determined using a standard curve of Quick Start Bovine Serum Albumin standards, also obtained from Bio-Rad.

## Protein Digestion

Sample concentration was adjusted to 10 μg protein in 8 M urea buffer. In order for the Lys-C pre-digestion, urea was diluted from 8 to 4 M by one volume of 20% acetonitrile, 100 mM AmBic as well as 0.1 μg Lys-C and then incubated in dark at 30°C for 5 h with 500 rpm (Niessen et al., 2006; Song and Liu, 2015).

For trypsin digestion, another volume of 10% Acetonitrile, 25 mM AmBic, 10 mM CaCl2 and 5 mM DTT was added to the sample resulting in a final urea concentration of 2M. After adding trypsin beads (Poroszyme, Applied Biosystems) samples were incubated overnight at 37°C. After centrifugation at 10,000 × g at 4°C supernatant was transferred into a new low-bind for desalting.

Agilent Bond Elut OMIX C18 pipette-based SPE stage tips from Agilent Technologies were used for desalting, according to the manufacturer's instructions and dried in a vacuum concentrator (ScanSpeed MaxiVac) and stored at −80°C until measurement.

## ESI LC-MS/MS Measurement

Protein digests were re-dissolved in 2% acetonitrile (ACN) and 0.1% formic acid (FA), ultra-sonicated for 15 s and subsequently centrifuged at 4°C for 10 min at 21,000 × g. An uHPLC system (Dionex Ultimate 3000) with a flow rate of 300 μl min^−1^ was used; and the column (Thermo scientific Easy Spray column) loaded with 1 μg protein performing a 145 min gradient from 2 to 90% ACN 0.1% FA. MS analysis with 21 scan events, 1 MS1 scan (FTMS) with a scan range of 350–1,800 m/z and 20 MS2 scans (ITMS) of the most abundant m/z ratios acquired from MS1 was carried out using a Thermo scientific velos rvo ion trap and Thermo scientific LTQ Orbitrap Elite. Default charge state was set to 2-fold charge; unassigned charge states as well as +1 charge states were rejected. Minimal required signal was set to 10,000, size of exclusion mass list was set to 500 (with a duration of 60 s) and exclusion mass width was set to 5 ppm with one repeated count of 30 s.

## Protein Identification and Quantification

Analysis of mass spectral data was performed using MaxQuant (1.5.3.8). Raw files were searched against a combined FASTA file for *Glycine max* and *Bradyrhizobum japonicum* with 82,928 entries (http://www.uniprot.org/ 21.10.2015). Tryptically digested peptides were allowed a maximum of 2 missed cleavages as well as a maximum of three modifications per peptide (oxidation, N-terminal acetylation). Precursor mass tolerance was set to 4.5 ppm (FTMS) and 0.6 Da (ITMS). To eliminate matching by chance, data was searched against a database of revert sequences in a target-decoy approach. Only high confidence peptides (FDR <0.01%) as well as proteins with at least two distinct identified peptides passed the criteria for identification. Additionally, the FDR based “matching between runs” algorithm was used (Cox and Mann, 2008). For relative quantification LFQ intensities were used.

Proteins were functionally categorized on basis of sequence similarity with proteins of other organisms via BLAST and RPS-BLAST against reference databases (ORYZA, PPAP, TAIR, KOG, CDD) using the Mercator sequence annotation tool (http://plabipd.de/portal/mercator-sequence-annotation) (Lohse et al., 2014). Proteins that could not be assigned to specific bins by Mercator were later manually assigned to categories according to protein name and functionality, or remained “not assigned.” A second BLAST was performed against UniRef100 database in order to find protein name and function for uncharacterized proteins contained in the original FASTA file. Tungsten responsive protein candidates were additionally validated on peptide level using ProtMax software for targeted identification/quantification of prototypic peptides (Egelhofer et al., 2013). The mass spectrometry proteomics data have been deposited to the ProteomeXchange Consortium via the PRIDE (Vizcaíno et al., 2016) partner repository with the dataset identifier PXD010877.

## Statistical Analysis and Data Mining

Statistical analysis of biomass and physiological data was performed with Infostat software (InfoStat, RRID:SCR_014310) (Di Rienzo et al., 2018). Biomass and physiological data was analyzed by one-way ANOVA using DGC *post hoc* test, if necessary heteroscedasticity was corrected. Translocation factor (TF) was calculated dividing nutrient and tungsten concentrations of shoots by root concentrations.

For statistical analysis of the protein data, only proteins detected in three or more replicates of at least one treatment were used. Missing values were imputed with the smallest value detected for each protein throughout all samples divided by two. An analysis of variance (ANOVA) followed by a Tukey HSD *post hoc* test, indicating a statistical significant difference at *p* < 0.05, was performed using R-studio. Averaged intensities for each treatment were used to calculate the ratios between control and high tungsten treatments. Proteins were considered significantly changing when they showed a ≥ 2-fold change as well as a *p*-value below 0.05. *P*-value correction was performed using Benjamini-Hochberg in order to account for false positives due to multiple testing, adjusted *P-*values are provided in the summary tables in the supplement.

Venn diagrams were made using the online tool Venny (version 2.1) (Oliveros, 2007). A principal component analysis (PCA) was performed for root and nodule proteins to additionally filter for effect size of identified proteins. The PCA was performed with R prcomp-package (Principal Component Analysis, RRID:SCR_014676), using the log2 transformed data, clustering was done using Euclidian distance with complete linkage (Team, 2017).

## Results

### Plant Growth and Nutrition

#### Biomass

**Figure 1:**
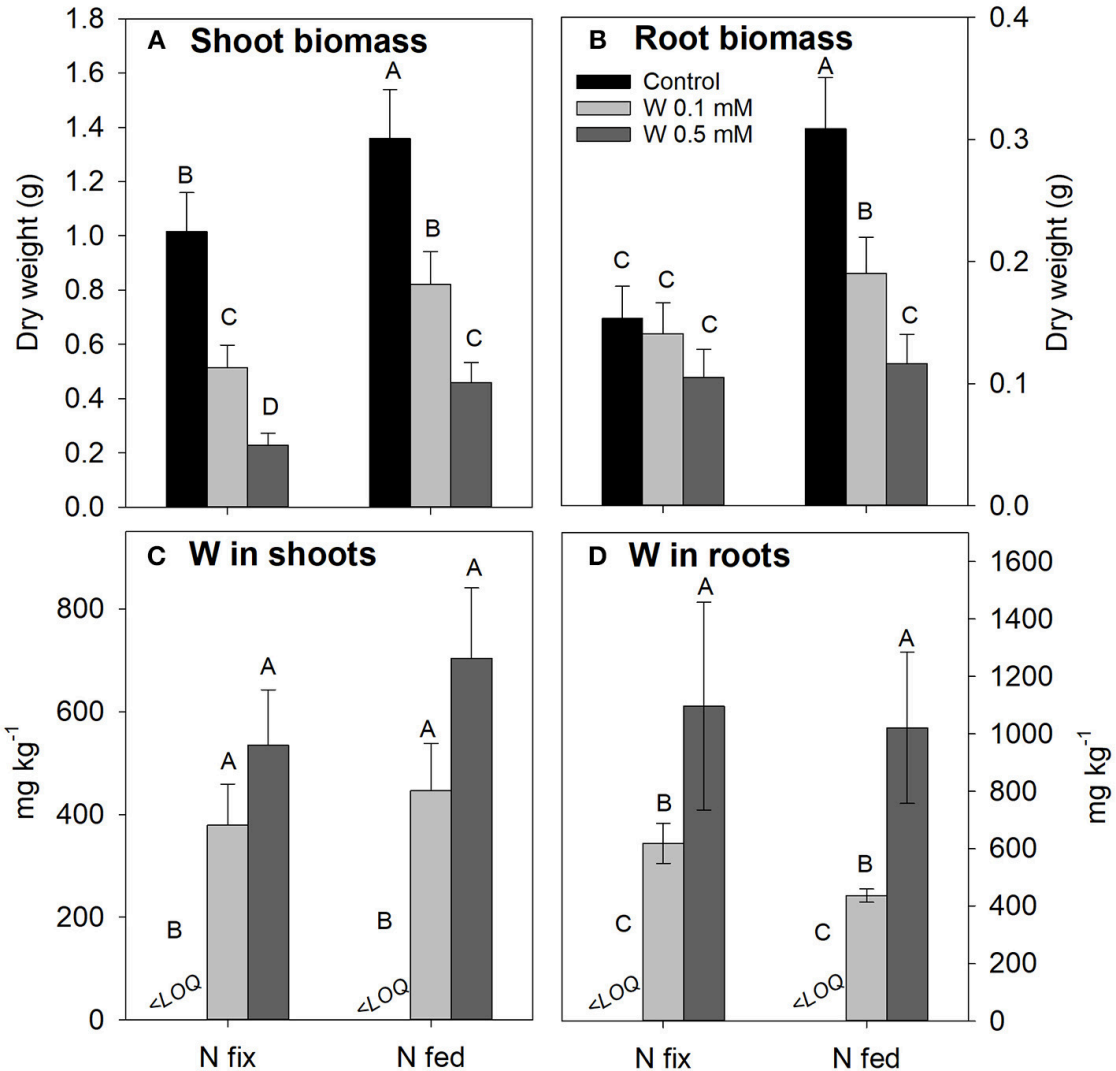
Shoot **(A)** and root **(B)** biomass as well as W tissue concentrations **(C,D)** of soybean (*Glycine max* cv Primus) grown semi-hydroponically with increasing W concentrations (control, 0.1, 0.5 mM W supplied as sodium tungstate) and under differing nitrogen supply regimes (N fix: week 2&3 0. 25 mM KNO_3_ & week 4–7 zero N; N fed: week 2–7 10 mM KNO_3_). Letters indicate significant differences across the different W and N treatments (*ANOVA, post hoc DGC, p* < 0.05). LOQ, limit of quantification.

Rising levels of W differentially affected shoot and root biomass of N_2_ fixing and N fed plants (Figures 1A,B). Shoot biomass of N_2_ fixing plants significantly decreased with increasing W concentrations, while root biomass remained comparable to the control treatment. The W induced decrease in shoot biomass production of N fed plants shows a similar decline as observed for N fix plants, however, N fed plants exhibited a significantly higher initial biomass for controls and throughout all tungsten treatments. Root biomass was more strongly affected in N fed compared to N_2_ fixing soybean, with concentrations of 0.1 and 0.5 mM W reducing root biomass of N fed soybean significantly compared to control as well as between the two tungsten treatments. Root biomass was overall lower in N_2_ fixing soybean plants and remained unchanged with increasing W concentrations. Root:shoot ratio showed a significant increase in N fix plants with increasing tungsten concentrations but did not significantly increase in the N fed treatment (Table S1). In both N regimes, plants exposed to 0.5 mM W only fully developed primary leaves while secondary and tertiary leaf generations were either missing or remained small (Figure S1A). In addition, roots showed blackening and exhibited a coral like morphology (Figure S1B).

#### W Concentrations

Tungsten concentrations in shoots were similar for both N and W treatments [ranging from 379 ± 80 (N fix; 0.1 mM W) to 703 ± 136 mg kg^−1^ (N fed; 0.5 mM W)], even though a non-significant increase in shoot W with increasing W exposure could be observed (Figure 1C). Tungsten concentration in roots significantly increased with increasing W levels, however, they did not differ between the different N treatments (Figure 1D). Estimates for nodule tungsten concentrations indicate that tungsten concentrations in nodules are slightly higher than in roots (Table S2). The root:shoot translocation factor was generally higher for N fed (1.02 (0.1 mM W); 0.87 (0.5 mM W) compared to N_2_ fixing plants [0.66 (0.1 mM W); 0.58 (0.5 mM W)] (Table S1).

#### N Acquisition

**Figure 2:**
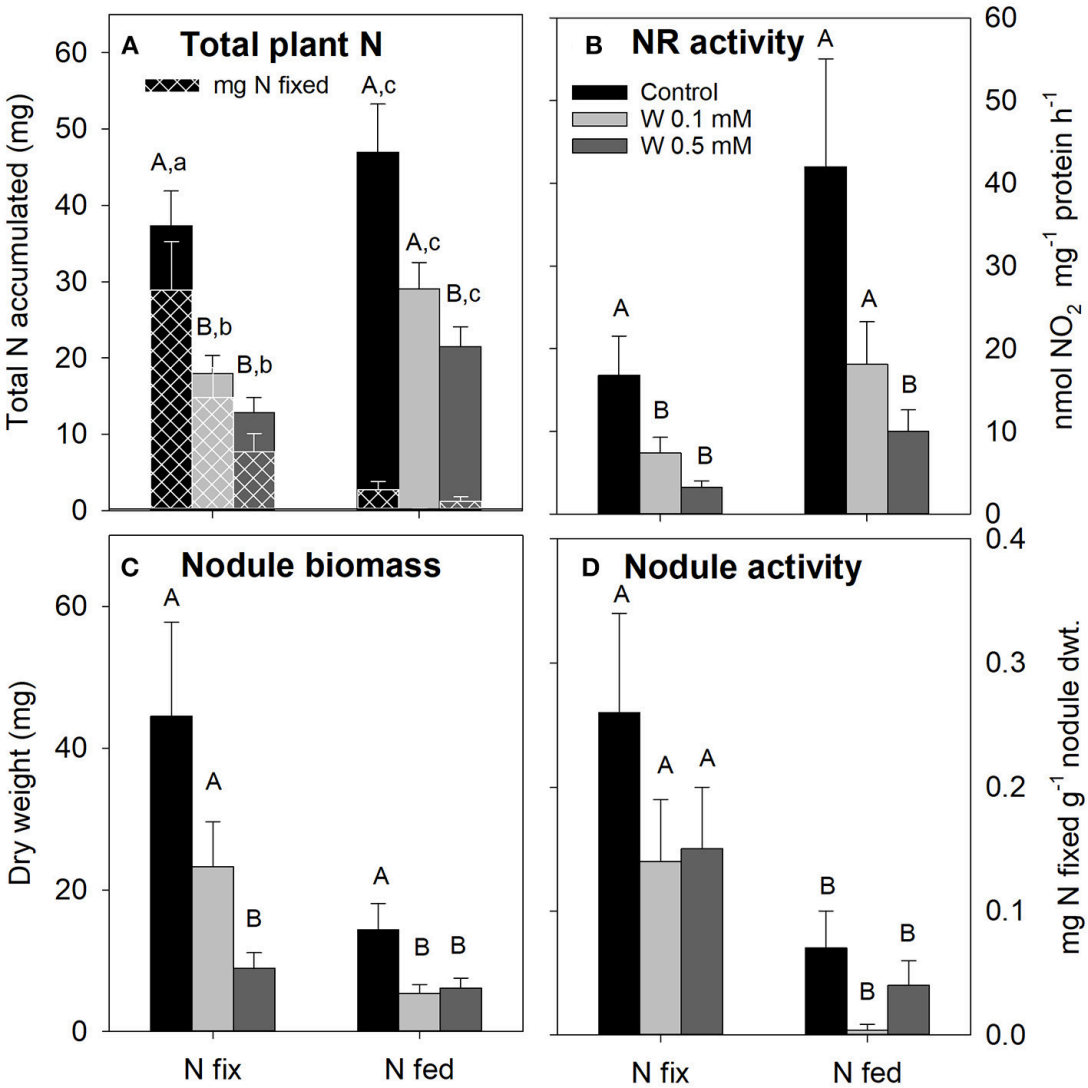
**(A)** Total nitrogen (N, mg) accumulated by soybean (*Glycine max* cv Primus) with the amount of N (mg) derived from symbiotic N_2_ fixation (mg N fixed) represented by the white checkered pattern. **(B)** Nitrate reductase activity in soybean leaves; **(C)** nodule biomass (mg); **(D)** nodule activity (mg N fixed g^−1^ nodule dry weight). Soybean was grown semi-hydroponically with increasing W concentrations (control, 0.1, 0.5 mM W supplied as sodium tungstate) and under differing nitrogen supply regimes (N fix: week 2&3 0. 25 mM KNO_3_ & week 4–7 zero N; N fed: week 2–7 10 mM KNO_3_). Small letters indicate significant differences of N derived from symbiotic N2 fixation, capital letters indicate significant differences of all other investigated parameters across the different W and N treatments (*ANOVA, post hoc DGC, p* < 0.05).

Increasing W concentrations also differentially affected plant N acquisition of N fix and N fed plants (Figure 2). Total plant N (i.e., N accumulated during the growth period) and NR activity were generally lower in N fix plants, however this was only statistically significant at 0.1 mM W. The total amount of plant N derived from symbiotic N_2_ fixation (mg N fixed, Figure 2A), nodule biomass (except plants grown at 0.5 mM W) and nodule activity on the other hand were overall higher in N fix plants compared to N fed. For N_2_ fixing plants, total plant N as well as NR-activity were significantly decreased at 0.1 mM W. While a further decline at 0.5 mM W was observed, this was not statistically significant for both parameters (Figures 2A,B). Nodule biomass also decreased but this was only significant at the highest W exposure (0.5 mM). Nodule activity of N fix plants on the other hand was reduced by approximately 50% (not significant) in both W treatments (Figures 2C,D) resulting in an average of 72 ± 12% of total plant N being derived from symbiotic N_2_ fixation irrespective of W concentration applied (Figure 2A). Despite a strong decrease, total accumulated N and NR-activity were only significantly affected at 0.5 mM W (Figures 2A,B). Nodule biomass was generally higher in N fix plants, but decreased with increasing W in both N regimes. Nodule activity was significantly lower in N fed plants and remained comparable to the respective control treatment a (Figures 2C,D). The contribution of symbiotically fixed N was <6% across all W treatments in N fed plants (Figure 2A).

#### Macro- and Micro-Nutrients

Concentrations of essential plant nutrients (and W) are shown in Table 1. In general, nitrogen fixing plants showed higher concentrations of macronutrients such as P, S, and Ca in shoot and root tissue and Mg in shoots only. Conversely K root and shoot concentrations were significantly higher in N fed plants. In shoot tissue, W had an increasing effect on Cr, Mo, and Fe in both N treatments; but while shoot concentrations of N_2_ fixing plants were already enhanced at 0.1 mM W, shoots of N fed plants only showed an increase in those micronutrients at the highest W addition (0.5 mM). While W significantly increased root P in both N treatments, shoot P was only enhanced by W in N fed plants. Shoot Mn and Mg was negatively affected by the highest W addition (0.5 mM) in both N treatments, Ca and K (N fix only) concentrations already decreased at 0.1 mM W.

**Table 1:** Shoot **(A)** and root **(B)** concentrations of tungsten, as well as macro- and micronutrients of soybean (*Glycine max* cv Primus) grown semi-hydroponically with increasing W concentrations (control, 0.1, 0.5 mM W supplied as sodium tungstate) and under differing nitrogen supply regimes (N fix: week 2&3 0. 25 mM KNO_3_ & week 4-7 zero N; N fed: week 2–7 10 mM KNO_3_).

	**unit**	**N fix**		**N fix**		**N fix**		**N fed**		**N fed**		**N fed**	
		**Control**		**0.1 mM W**		**0.5 mM W**		**Control**		**0.1 mM W**		**0.5 mM W**	
**(A) SHOOT**													
**W**	mg kg^−1^	< LOQ	**B**	379 ± 80.1	**A**	535 ± 108	**A**	< LOQ	**B**	446 ± 92.2	**A**	704 ± 137	**A**
**Mo**	mg kg^−1^	2.12 ± 0.82	**B**	5.14 ± 2.11	**A**	18.60 ± 8.28	**A**	1.32 ± 0.50	**B**	1.95 ± 0.75	**B**	10.28 ± 4.41	**A**
**Cu**	mg kg^−1^	9.5 ± 1.75	**A**	2.6 ± 2.47	**B**	11.5 ± 1.7	**A**	6.4 ± 1.95	**B**	4.8 ± 2.10	**B**	6.5 ± 1.94	**B**
**Cr**	mg kg^−1^	3.4 ± 1.06	**B**	13.2 ± 4.86	**A**	40.8 ± 17.0	**A**	2.2 ± 0.66	**B**	2.7 ± 0.82	**B**	22.0 ± 8.6	**A**
**Mn**	mg kg^−1^	66.5 ± 9.94	**A**	59.1 ± 9.27	**A**	27.8 ± 5.92	**B**	42.5 ± 7.62	**B**	31.66 ± 6.4	**B**	26.4 ± 5.74	**B**
**Fe**	mg kg^−1^	220 ± 68.9	**B**	667 ± 218	**A**	774 ± 255	**A**	200 ± 62.4	**B**	126 ± 38.5	**B**	575 ± 187	**A**
**P**	g kg^−1^	3.79 ± 0.30	**A**	3.94 ± 0.32	**A**	3.81 ± 0.30	**A**	1.82 ± 0.07	**C**	2.14 ± 0.10	**B**	2.25 ± 0.11	**B**
**Mg**	g kg^−1^	7.59 ± 0.26	**A**	7.30 ± 0.26	**A**	4.74 ± 0.26	**C**	6.53 ± 0.26	**B**	6.63 ± 0.26	**B**	4.56 ± 0.26	**C**
**S**	g kg^−1^	5.63 ± 0.78	**A**	6.80 ± 1.05	**A**	4.16 ± 0.48	**A**	2.49 ± 0.21	**B**	4.85 ± 0.61	**A**	4.91 ± 0.62	**A**
**Ca**	g kg^−1^	7.13 ± 0.21	**A**	5.83 ± 0.20	**B**	2.62 ± 0.17	**D**	4.2 ± 0.19	**C**	5.9 ± 0.20	**B**	2.91 ± 0.17	**D**
**K**	g kg^−1^	26.1 ± 1.40	**C**	22.1 ± 1.06	**D**	17.20 ± 0.70	**E**	35.7 ± 2.36	**B**	48.3 ± 3.91	**A**	37.1 ± 2.52	**B**
**(B) ROOT**													
**W**	mg kg^−1^	< LOQ	**B**	619 ± 70.0	**A**	1,097 ± 361	**A**	< LOQ	**B**	436 ± 23.2	**A**	1,021 ± 263	**A**
**Mo**	mg kg^−1^	5.39 ± 1.27	**A**	2.47 ± 1.69	**A**	7.19 ± 2.31	**A**	2.21 ± 1.07	**A**	3.48 ± 1.05	**A**	4.88 ± 2.23	**A**
**Cu**	mg kg^−1^	14.2 ± 4.49	**B**	11.9 ± 4.48	**B**	72.1 ± 14.3	**A**	20.7 ± 5.71	**B**	32.7 ± 7.67	**B**	36.4 ± 8.22	**B**
**Cr**	mg kg^−1^	33.4 ± 7.21	**A**	25.1 ± 4.85	**A**	56.9 ± 20.7	**A**	14.6 ± 1.66	**B**	24.9 ± 4.28	**A**	50.9 ± 15.2	**A**
**Mn**	mg kg^−1^	48.2 ± 5.98	**B**	56.9 ± 7.91	**B**	60.2 ± 8.39	**B**	42.1 ± 5.20	**B**	101 ± 12.8	**A**	52.9 ± 6.57	**B**
**Fe**	g kg^−1^	2.20 ± 0.34	**A**	1.27 ± 0.16	**B**	2.12 ± 0.36	**A**	0.95 ± 0.09	**B**	1.02 ± 0.10	**B**	1.43 ± 0.17	**B**
**P**	g kg^−1^	2.56 ± 0.17	**C**	4.64 ± 0.44	**A**	3.71 ± 0.31	**B**	1.58 ± 0.08	**D**	3.16 ± 0.24	**B**	3.50 ± 0.28	**B**
**Mg**	g kg^−1^	5.54 ± 0.56	**A**	2.95 ± 0.39	**B**	2.31 ± 0.35	**B**	5.65 ± 0.57	**A**	4.06 ± 0.45	**A**	2.93 ± 0.38	**B**
**S**	g kg^−1^	6.03 ± 0.44	**A**	7.14 ± 0.55	**A**	2.89 ± 0.18	**C**	6.28 ± 0.47	**A**	6.27 ± 0.46	**A**	3.80 ± 0.25	**B**
**Ca**	g kg^−1^	7.99 ± 0.80	**B**	9.73 ± 0.61	**B**	9.69 ± 0.61	**B**	13.5 ± 0.34	**A**	10.3 ± 0.56	**B**	9.98 ± 0.58	**B**
**K**	g kg^−1^	15.6 ± 1.44	**C**	19.5 ± 1.80	**B**	8.32 ± 0.77	**D**	38.7 ± 3.55	**A**	33.1 ± 3.04	**A**	13.4 ± 1.24	**C**

*Letters indicate significant differences across the different W and N treatments (ANOVA, Post Hoc DGC, p < 0.05). LOQ - limit of quantification*.

#### Starch Content

**Figure 3:**
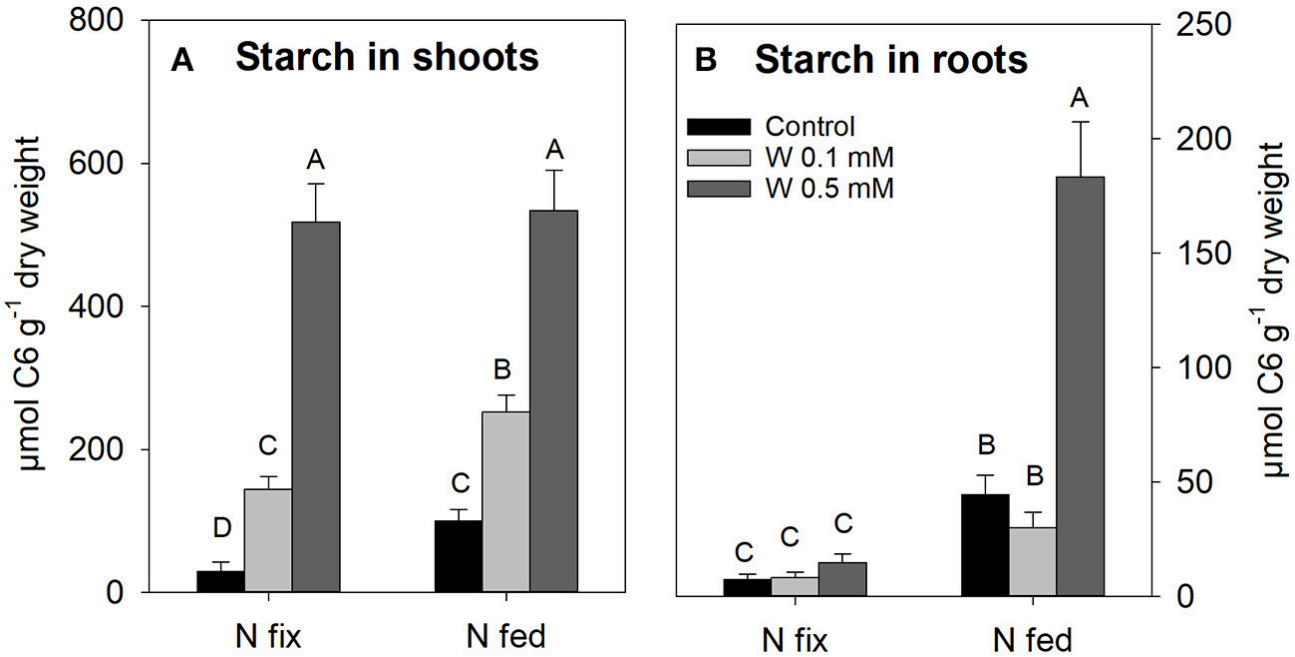
Starch concentration (μmol C6 g^−1^ dry weight) in shoots **(A)** and roots **(B)** of soybean (*Glycine max* cv Primus) grown semi-hydroponically with increasing W concentrations (control, 0.1, 0.5 mM W supplied as sodium tungstate) and under differing nitrogen supply regimes (N fix: week 2&3 0. 25 mM KNO_3_ & week 4–7 zero N; N fed: week 2–7 10 mM KNO_3_). Letters indicate significant differences across the different W and N treatments (*ANOVA, post hoc DGC, p* < 0.05).

Shoot starch concentrations significantly increased with increasing W levels in both N treatments; however, starch concentrations were generally lower in the control and 0.1 mM W treatments of N fixing plants when compared to the respective N fed plants (Figure 3A). In N_2_ fixing plants, starch in roots remained low with increasing W exposure, while starch in roots of N fed plants was higher than in N_2_ fixing plants and strongly increased at 0.5 mM W by an average factor of 5±1 compared to the control and 0.1 mM W treatment (Figure 3B).

## Tungsten Induced Proteomic Changes

### Proteomic Analysis of Roots and Nodules

Overall, of 2,479 identified proteins, 2,096 were detected in at least three out of five replicates of at least one treatment. The analysis of variance followed by a Tukey honest significant difference *post hoc* test revealed that 678 of the 2,096 identified proteins showed a significantly different regulation between W stressed plants and corresponding control, in at least one organ and one N regime (*p* = 0.05). A set of 156 proteins was significantly changed between the two nitrogen treatments (*p* = 0.05) irrespective of W concentration applied. A full list of significantly affected proteins can be found in the supplements (Tables S4, S5).

### Root Proteome

Overall 375 root-proteins significantly changed due to tungsten addition, with an almost equal amount of proteins changing in both modes of nitrogen supply as well as in each group exclusively (N fed 34.1%, N fix 32% and 33.9% in both) (Figure S2, Table S4).

**Figure 4:**
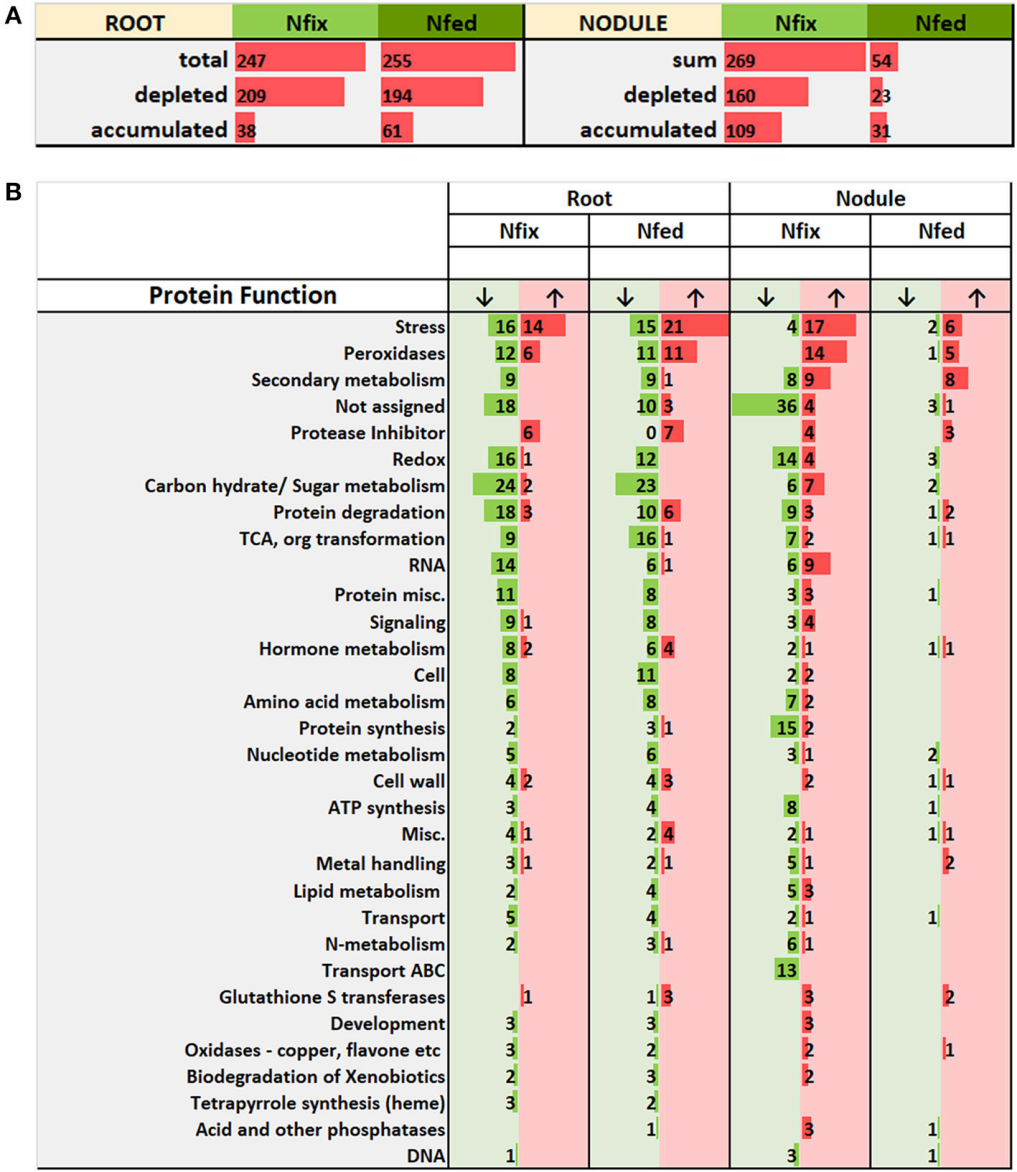
**(A)** Number of root and nodule proteins significantly (*ANOVA, post hoc Tukey, p* < 0.05) accumulated (↑) and depleted (↓), comparing control with high W (0.5 mM W, supplied as sodium tungstate) of each nitrogen regime (N fix: week 2&3 0. 25 mM KNO_3_ & week 4–7 zero N; N fed: week 2–7 10 mM KNO_3_). **(B)** shows a detailed list of affected molecular functions. “Misc.” includes categories with < 5 proteins (Co-factor and vitamin metabolism, GDSL-motif lipase, myrosinases-lectin-jacalin, misc. nitrilases (nitrile lyases, berberine bridge enzymes, reticuline oxidases, troponine reductases) troponine reductases, S-assimilation, short chain dehydrogenase/reductase (SDR), plastocyanin-like); “Carbon hydrate/ Sugar metabolism” includes functional categories involved in sugar and carbon metabolic processes (Oxidative pentose phosphate pathway, beta 1,3 glucan hydrolases, glucan endo-1,3-beta-glucosidase, major and minor CHO metabolism, Gluco-, galacto- and mannosidases, C1-metabolism, Glycolysis).

**Figure 5:**
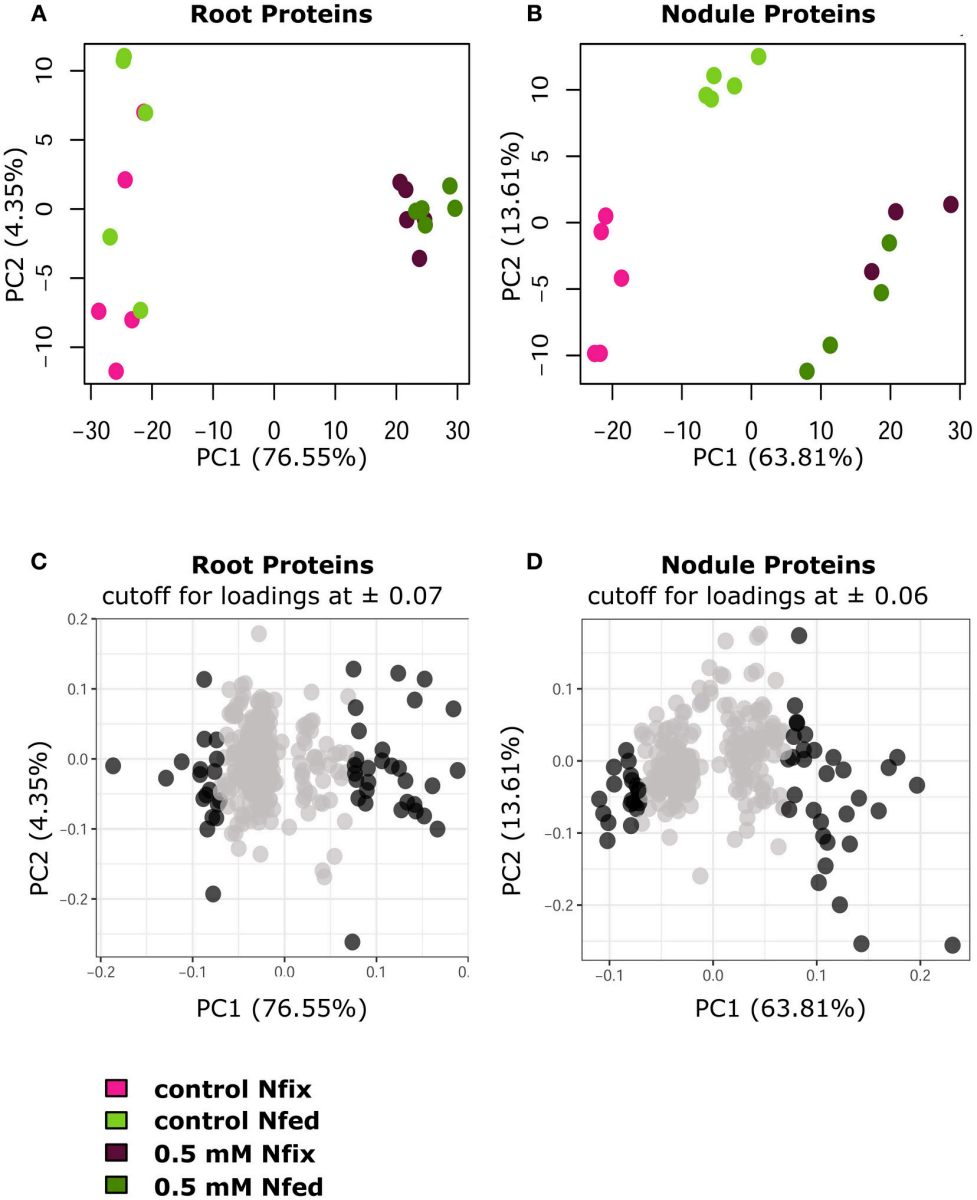
PCA plots (PC1/PC2 of log2 protein LFQ intensities) of significantly changed root **(A)** and nodule **(B)** proteins (*ANOVA, post hoc Tukey, p* < 0.05), comparing control with 0.5 mM W (supplied as sodium tungstate) of each nitrogen regime (N fix: week 2&3 0.25 mM KNO_3_ & week 4–7 zero N; N fed: week 2–7 10 mM KNO_3_). **(C,D)** PCA loadings of individual proteins (PC1 plotted against PC2) with a cutoff for root proteins **(C)** at 0.07, and a cutoff for nodule proteins **(D)** at 0.06 for PC1. Proteins outside of the determined cutoff are represented by dark gray dots.

**Figure 6:**
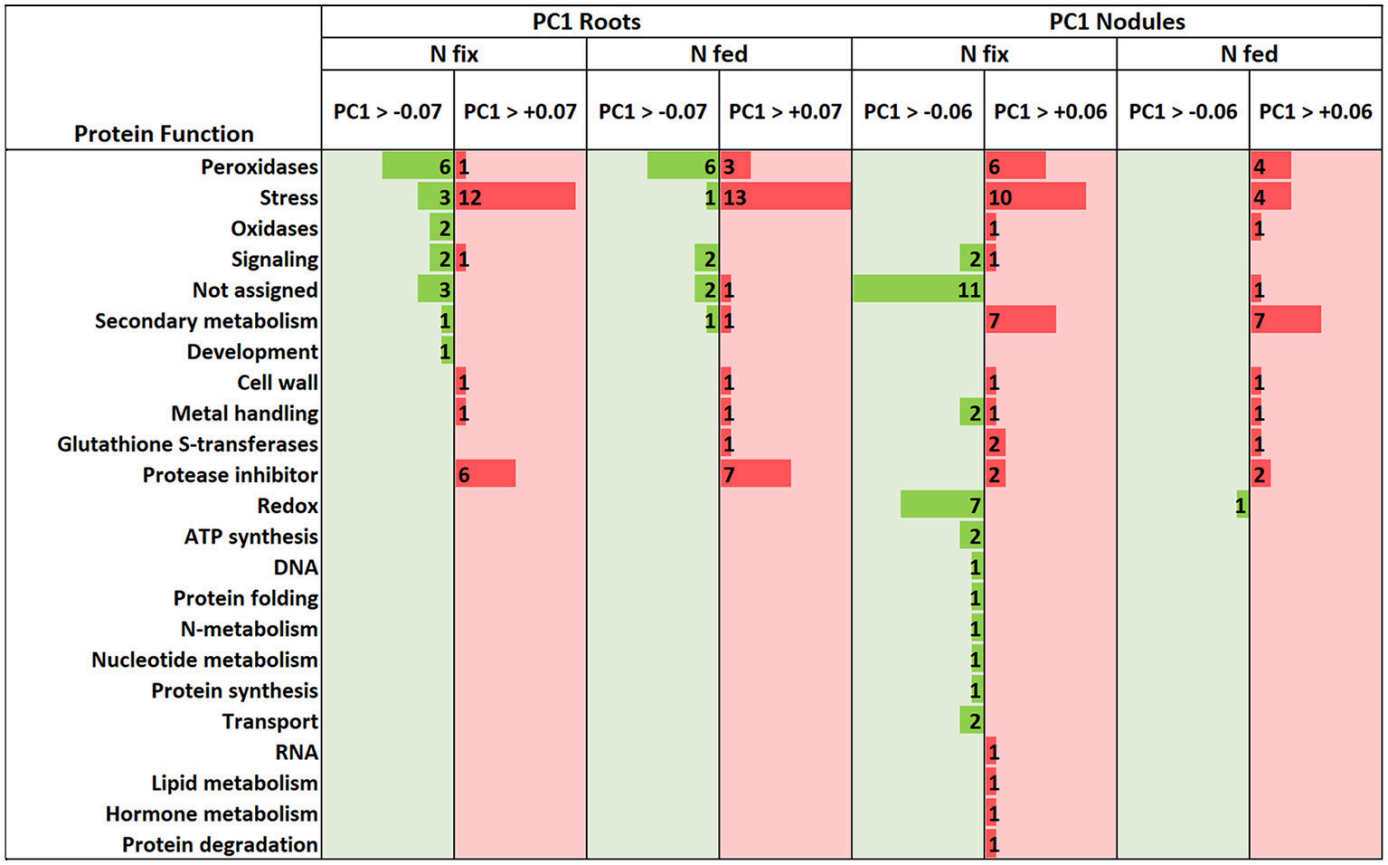
Molecular function of proteins above and below the cutoff for significantly (*ANOVA, post hoc Tukey, p* < 0.05) changed root and nodule proteins that were responsible for the variance (PC1, see Figure 5A –roots & Figure 5B-nodules) between control and high W (0.5 mM W, supplied as sodium tungstate).

Generally, exposure to high W concentrations (0.5 mM) lead to a depletion of protein abundances in roots of both N treatments (Figure 4A). Of the 247 proteins changed in N fix plants, 209 (i.e., 85%) showed a depletion while only 38 proteins accumulated. In the N fed treatment, 194 of the 255 changed proteins (i.e., 76%) were significantly depleted and 61 showed an increase in abundance. Functional categories that were significantly affected by the presence of tungsten are shown in Figure 4B. The root PCA shows a clear separation between control treatment (zero W) and high tungsten concentrations independent of N regime (0.5 mM W) through PC1, which was responsible for 76.6% of the variance (Figure 5A, Table S6B). PC2 only contributed 4.35% to the variance and neither lead to a separation between the different tungsten concentrations, nor between nitrogen fixing (N fix) and nitrogen fed (N fed) plants. In order to identify the proteins responsible for this separation, a threshold for proteins with the largest negative and positive loadings was set at −0.07 and 0.07 (Figure 5C). The PCA revealed that proteins involved in response to stress (germin-like proteins, thaumatin-like proteins, starvation associated messenger (SAM-22), protein P21, pathogenesis-related protein 10, chitinases) (Figure S4), peroxidases as well as protease inhibitory proteins (trypsin inhibitors, alpha amylase/subtilisin inhibitors) contributed most to PC1 and thus to a differentiation between the different tungsten treatments (Figure 6).

### Nodule Proteome

The majority (82.2%) of the 303 proteins affected by tungsten addition (0.5 mM), exclusively changed in nitrogen fixing plants (Figure S2). Only 20 (6.6%) were affected in both nitrogen treatments; 34 proteins (11.2%) exclusively changed in nodules of nitrogen fed plants (Figure 4). Of overall 269 proteins that significantly changed in nodules of the N fix treatment, 109 showed an increase, and 160 were significantly decreased at high levels of tungsten (Figure 4A, Table S5). Functional categories that were significantly affected by the presence of tungsten are shown in Figure 5B. Principal component analysis revealed that PC1 is responsible for 63.8% of the variation and separates the nodule proteins between control and high tungsten independent of N regime (Figure 5B). PC2 (13.61%) additionally separates N fix control from N fed control and the 0.5 mM W treatments. To identify the proteins most responsible for the observed separation, a cutoff of +0.06 and −0.06 was set for PC1 loadings (Figure 5D, Table S6A). The largest negative loadings for PC1 can be attributed to proteins lower in abundance in control treatments compared to plants exposed to W (Figure 6). The majority (21) of the 30 proteins with the largest negative loadings below said cutoff were rhizobia proteins depleted in high tungsten treatments as well as in control treatments of N fed plants. Figure 6 further shows that, after proteins without assigned function, especially “redox” (Leghemoglobin [A,C1,C2,C3], SOD, glutaredoxin), “metal handling” (Ferritin), “signaling” (nitrogen regulatory protein PII) and “mitochondrial electron transport, ATP synthesis” (Electron transfer flavoprotein, cytochrome b-c1 complex, ATP synthase) as well as “N-metabolism” (nitrogen fixation protein) and “transport ABC” (Amino acid ABC transporter ATP-binding) negatively contribute to PC1. The largest positive loadings can be attributed to proteins that were increased in presence of tungsten. Functional categories “stress” (Thaumatin-like protein, germin-like proteins, stress-induced proteins SAM22, probable protein P21, mRNA from stress-induced gene (H4), 18.5 kDa class heat shock protein) (Figure S4), “secondary metabolism” (Chalcone—flavonone isomerase 1A/1B-1, NAD(P)H-dependent 6'-deoxychalcone synthase, isoflavone reductase like), “peroxidases,” “endopeptidase inhibitor” (putative Kunitz-trypsin protease inhibitor), “protein degradation” (Subtilisin-like protease), as well as “hormone metabolism” (linoleate 9s-lipoxygenase) and “glutathione S-transferases” are positively contributing to PC1 (Figures 5D, 6).

### Overall Response

Of 621 significantly changed proteins, 57 proteins changed in roots and nodules upon 0.5 mM W exposure compared to the control treatments, irrespective of N regime (Figure S3A). Of protein changed in both roots and nodules, functional categories “stress,” “peroxidases,” “secondary metabolism,” “protein degradation,” and “endopeptidase inhibitor” were most affected (Figure S3A). Additionally, proteins of “TCA, organ transformation,” “cell wall,” “glutathione S-transferases,” “glycolysis” and “metal handling” and others (Figure S3B) significantly changed in both organs. Of these Proteins 5 changed in both organs and both N regimes between control and high tungsten (Table S3).

Of the 57 proteins that changed in roots and nodules, 14 also contributed to the principle component responsible for a separation between the different tungsten treatments (PC1) of their respective organs (Figure 6). Twelve of these proteins showed same accumulation patterns and were significantly changed in at least three of four combinations (Table 2). These proteins can be assigned to the functional categories “cell wall,” “protease inhibitors,” “stress,” “peroxidases,” and “metal handling.”

**Table 2:** List of significantly changed proteins in both organs (roots, nodules) and N regimes (N fix: week 2&3 0. 25 mM KNO_3_ & week 4–7 zero N; N fed: week 2–7 10 mM KNO_3_) between control and high tungsten (0.5 mM W, supplied as sodium tungstate) that show the same trend and were included in PC1 of their respective organ.

					**N fix**						**N fed**					
**ORGAN**	**Uniprot ID**	**Proteotypic evaluation**	**Mercator categories**	**Annotation (UniprotKB/Uniref100)**	**ztrans LFQ int**.						**ztrans LFQ int**.					
					**C**	**W 500**	**ratio**	***p*-value**	**BH adj. *p*-value**	**sig**.	**C**	**W 500**	**ratio**	***p*-value**	**BH adj. *p*-value**	**sig**.
ROOT	C6SYB8	x	stress	Stress-basic secretory protein PR	−0.8	0.5	0.08	0.02	0.12	*	−0.8	1.2	0.06	0.00	0.01	***
NODULE	C6SYB8	x	stress	Stress-basic secretory protein PR	−1.1	1.0	0.06	0.04	0.19	*	−0.6	0.7	0.30	0.19	1.00	
ROOT	C6T3A2		stress	SAM22	−0.8	0.6	0.21	0.02	0.12	*	−0.9	1.1	0.13	0.00	0.02	***
NODULE	C6T3A2		stress	SAM22	−0.7	1.4	0.00	0.05	0.23	*	−0.7	0.1	0.03	0.66	1.00	
ROOT	C7S8D4		stress	Germin-like protein subfamily 1 member 7	−0.9	1.0	0.01	0.00	0.03	**	−0.9	0.7	0.01	0.01	0.07	**
NODULE	C7S8D4		stress	Germin-like protein subfamily 1 member 7	−0.7	1.4	0.17	0.02	0.13	*	−0.7	0.0	0.36	0.56	1.00	
ROOT	I1J7M4	(X)	peroxidases (GST)	Peroxidase P7	−0.8	0.5	0.02	0.10	0.33		−0.8	1.2	0.01	0.01	0.06	**
NODULE	I1J7M4	(X)	peroxidases (GST)	Peroxidase P7	−0.8	1.3	0.04	0.00	0.00	***	−0.7	0.2	0.15	0.01	0.50	*
ROOT	I1K6M2	(X)	stress	Protein P21-like	−0.8	1.0	0.05	0.00	0.03	**	−0.9	0.8	0.02	0.00	0.04	**
NODULE	I1K6M2	(X)	stress	Protein P21-like	−0.8	1.4	0.08	0.01	0.08	**	−0.7	0.1	0.21	0.42	1.00	
ROOT	I1KW54		protease inhibitor	Kunitz-trypsin inhibitor	−0.8	0.7	0.02	0.01	0.10	*	−0.9	1.1	0.01	0.00	0.03	**
NODULE	I1KW54		protease inhibitor	Kunitz-trypsin inhibitor	−0.8	0.7	0.10	0.07	0.28		−0.9	1.0	0.02	0.01	0.39	**
ROOT	I1L927		stress	Thaumatin-like protein 1a	−0.9	1.1	0.00	0.00	0.05	**	−0.9	0.6	0.00	0.02	0.15	*
NODULE	I1L927		stress	Thaumatin-like protein 1a	−0.7	1.4	0.13	0.00	0.02	**	−0.7	0.0	0.33	0.35	1.00	
ROOT	I1LEI5		stress	Stress-basic secretory protein PR	−0.9	0.9	0.07	0.01	0.06	**	−0.9	0.8	0.07	0.01	0.08	**
NODULE	I1LEI5		stress	Stress-basic secretory protein PR	−1.0	0.3	0.15	0.23	0.60		−0.6	1.3	0.23	0.03	0.71	*
ROOT	I1MI59	x	protease inhibitor	Kunitz-trypsin inhibitor	−0.9	0.9	0.02	0.00	0.00	***	−0.9	0.8	0.01	0.00	0.00	***
NODULE	I1MI59	x	protease inhibitor	Kunitz-trypsin inhibitor	−0.8	1.3	0.00	0.00	0.00	***	−0.8	0.3	0.01	0.00	0.04	***
ROOT	I1MV71	x	cell wall	Expansin-like B1	−0.8	0.4	0.01	0.00	0.00	***	−0.8	1.2	0.00	0.00	0.00	***
NODULE	I1MV71	x	cell wall	Expansin-like B1	−0.8	1.3	0.03	0.00	0.00	***	−0.8	0.3	0.02	0.00	0.00	***
ROOT	K7KJM5	x	metal handling	Polyphenol oxidase	−0.9	0.7	0.07	0.01	0.08	**	−0.8	1.0	0.08	0.00	0.03	**
NODULE	K7KJM5	x	metal handling	Polyphenol oxidase	−0.7	0.0	0.33	0.70	1.00		−0.7	1.4	0.15	0.02	0.65	*
ROOT	Q9XFI8		peroxidases	Peroxidase 4	−0.8	0.3	0.09	0.10	0.32		−0.8	1.3	0.04	0.00	0.02	***
NODULE	Q9XFI8		peroxidases	Peroxidase 4	−0.9	0.6	0.07	0.00	0.02	***	−0.8	1.1	0.09	0.00	0.02	***

*Asterisk indicate significant difference (ANOVA, post hoc Tukey, p < 0.05). Proteins uniquely identified and quantified by prototypic peptides are indicated with an x. Proteins uniquely quantified by prototypic peptides are indicated with an (x). When protein identification and quantification was based on non-proteotypic peptides, the Uniprot ID represents the majority protein accession of a protein group*.

## Nitrogen Regime-Dependent Differences

### Control Treatments (C-N Fix vs. C-N Fed)

Of 127 proteins changed in the zero tungsten control treatments upon different N regimes (N fix, N fed) (Tables S8A,B), 54 were constitutively elevated in the nitrogen fed plants. These proteins can be primarily assigned to “peroxidases,” “CHO metabolism” (“glycolysis,” “major” and “minor CHO metabolism,” “gluco-, galacto-, mannosidases,” “OPP”) “stress,” “not assigned,” “glutathione s transferases” and “cell wall,” as well as “redox” and “cell.” Slightly more (73) proteins were less abundant upon nitrate addition, with the majority (54 proteins) of these proteins being rhizobial proteins. Many of these proteins have still unknown function and thus result in “not assigned” being the largest category. Further functional categories that were significantly decreased are “protein” (“protein synthesis,” “protein folding,” “protein posttranslational modification”), “redox” (Leghemoglobin (A, C1, C2, C3), glutaredoxin, superoxide dismutase), “CHO metabolism” (sugar hydrolase, oligosaccharide deacetylase, etc.), “metal handling” and “amino acid metabolism” (see Tables S8A,B).

### Tungsten Treatment (0.5 mM W-N Fix vs. 0.5 mM W-N Fed)

Only a small set of 32 proteins significantly changed between the two high tungsten treatments upon differing N supply (Tables S8A,B). Proteins increased due to nitrate addition (N fed) and high W were assigned to the functional categories “RNA” and “protein degradation” but also “peroxidases,” “protein,” “amino acid metabolism,” “nucleotide metabolism,” “lipid metabolism,” “N-metabolism” and “myrosinases-lectin-jacalin” as well as “not assigned.” Proteins only accumulating in N_2_ fixing plants exposed to high W were assigned to “stress,” “peroxidases,” “acid and other phosphatases,” “hormone metabolism,” “signaling,” “metal handling,” “glutathione s transferases” (see Tables S8A,B).

## Discussion

### Tungsten Differentially Hampers Growth and Development of N Fix and N Fed Soybean

The high dose of nitrate supplied (10 mM) to N fed plants resulted in an overall higher biomass of N fed compared to N fix plants irrespective of W addition. Depending on cultivar and growth conditions, the majority of soybean N demand can be covered by biological N_2_ fixation, a certain amount of N is usually derived from naturally occurring soil N (Salvagiotti et al., 2008). Thus, while the continuously administered dose of NO_3_ in N fed plans resulted in enhanced growth of N fed control plants, the initial dose of 0.25 mM NO_3_ was apparently not providing additional growth in the N fix treatment.

Increasing W concentrations in the nutrient solution differentially affected root and shoot biomass of N fix and N fed soybean plants, despite similar W tissue concentrations within each W application level (Figures 1A,B). While root biomass of N fix plants was not affected, root biomass of N fed plants significantly decreased with increasing W concentrations (Figures 1A,B). This suggests that under W induced stress, a strong dependency of rhizobial activity (N fix plants) results in a change in resource allocation that allowed maintenance of root biomass to ensure symbiosis functioning and plant growth. The size of below ground biomass is crucial for maintenance of nutrient uptake during adverse condition and can have beneficial effects on plant health during stress (Glick, 2003). This is supported by the fact that despite reduced nodulation (0.5 mM W), N_2_ fixation (nodule activity) of N fix soybean was not affected by increasing W concentrations (Figure 2D).

Our results correspond well with findings of Harper and Nicholas (1978), who reported a significantly decreased biomass production (~30% of the control) of 21 day old soybean plants grown on 0.4 mM W. While in non-symbiotically grown *Pisum sativum* Adamakis et al. (2008) found reduced root length at 200 and 500 mg L^−1^ sodium tungstate (329.85 g mol^−1^) after 8 days of growth, Jiang et al. (2004) already found significant reduction of organ length at concentrations higher than 10 μM sodium tungstate in *Hordeum vulgare* after 9 days of growth.

The necrotic blackening of roots (typically derived from oxidation of phenolic compounds) and a coral like morphology due to reduced side root development of soybean at 0.5 mM W (Figure S1) in both N regimes corresponded well with findings of other studies investigating W phytotoxicity in plant species such as oat, radish and lettuce (Bamford et al., 2011; Adamakis et al., 2012). Similar morphological changes have also been described for lead and arsenic treated soybeans (Békésiová et al., 2008) suggesting similar metal toxicity response mechanisms for W as for other heavy metals.

### Tungsten Impedes Nitrate Assimilation and Nodule Functionality Differentially

At high tungsten concentrations (0.5 mM), not only above ground biomass of both nitrogen treatments were negatively affected, NR activity also dropped to only 20% of the activity of healthy N fed plants (Figure 2B). This does not come to surprise, since it has been shown that, due to the competition with molybdenum as enzymatic co-factor, tungsten leads to a decreased NR activity but does not inhibit its synthesis (Heimer et al., 1969; Deng et al., 1989; Adamakis et al., 2012; Xiong et al., 2012). Harper and Nicholas (1978) found that while nitrate reductase activity of N fed soy plants (6 mM KNO_3_) continuously decreased with increasing tungsten levels to 8.6% at 0.4 mM compared to control plants (23% at 0.5 mM in our experiment), acetylene reduction showed a 3-fold increase between control and 0.4 mM. The authors suggested that the observed patterns together with an increase in nodule biomass were an effect of the inhibition of the NO${}_{3}^{-}$ metabolism by W resulting in an increase in C:N ratio (Harper and Nicholas, 1978). Such a compensation of NR activity by increased nodulation and enhanced N_2_ fixation activity of soy was also observed in one of our previous studies investigating bioavailability, speciation and phytotoxicity of tungsten in soil (Oburger et al., 2018). In the present study, however, no increase or compensation of N_2_-fixation was observed, possibly since the higher dose of NO_3_ of N fed plants (we compared 10 mM KNO_3_ fed plants with plants receiving only 0.25 mM KNO_3_ for the first 2 weeks of growth) already impeded nodule formation and thus N_2_-fixation (Kanayama et al., 1990; Salvagiotti et al., 2008).

Unlike nitrate reductase, the effect of tungsten on bacterial nitrogen fixation (BNF) via nitrogenase seems not to be simply explainable by the inhibition of catalytic function of the enzyme by substitution of Mo with W, since the N_2_ fixation activity (mg N fixed g^−1^ nodule dwt.) of the fewer nodules formed under high W was not fully inhibited by tungsten (~55% activity remained at 0.5 mM W) (Figure 2D). Consistent with that, some studies found indices for an active nitrogenase even in presence of tungsten and that inhibitory effects of tungsten on nitrogenase are more pronounced in Mo deficient conditions (Kletzin and Adams, 1996; Schwarz et al., 2007; Ringelberg et al., 2009; Strigul et al., 2009). In *Rhodospirillum rubrum* it was shown that, although lower than activity in Mo grown cells, nitrogenase activity (measured via H_2_ evolution) was not inhibited by W addition (Paschinger, 1974). Nagatani and Brill (1974) found that in *Azotobacter Vinelandii* specific activity of nitrogenase component one (NifD and NifK) but not component II was reduced in Mo-free, W containing medium. Furthermore, they found that relative amounts of component I protein were still present in W grown cultures (56% of normal component I protein) and that the activity of the component was reinstated 3 h after the addition of Mo to the medium. More recently, however, studies on *Rhodobacter capsulatus* suggest that, although possessing proton reduction activity, tungsten substituted nitrogenase is incapable of N_2_ fixation and acetylene reduction (Siemann et al., 2003; Schwarz et al., 2007).

**Figure 7:**
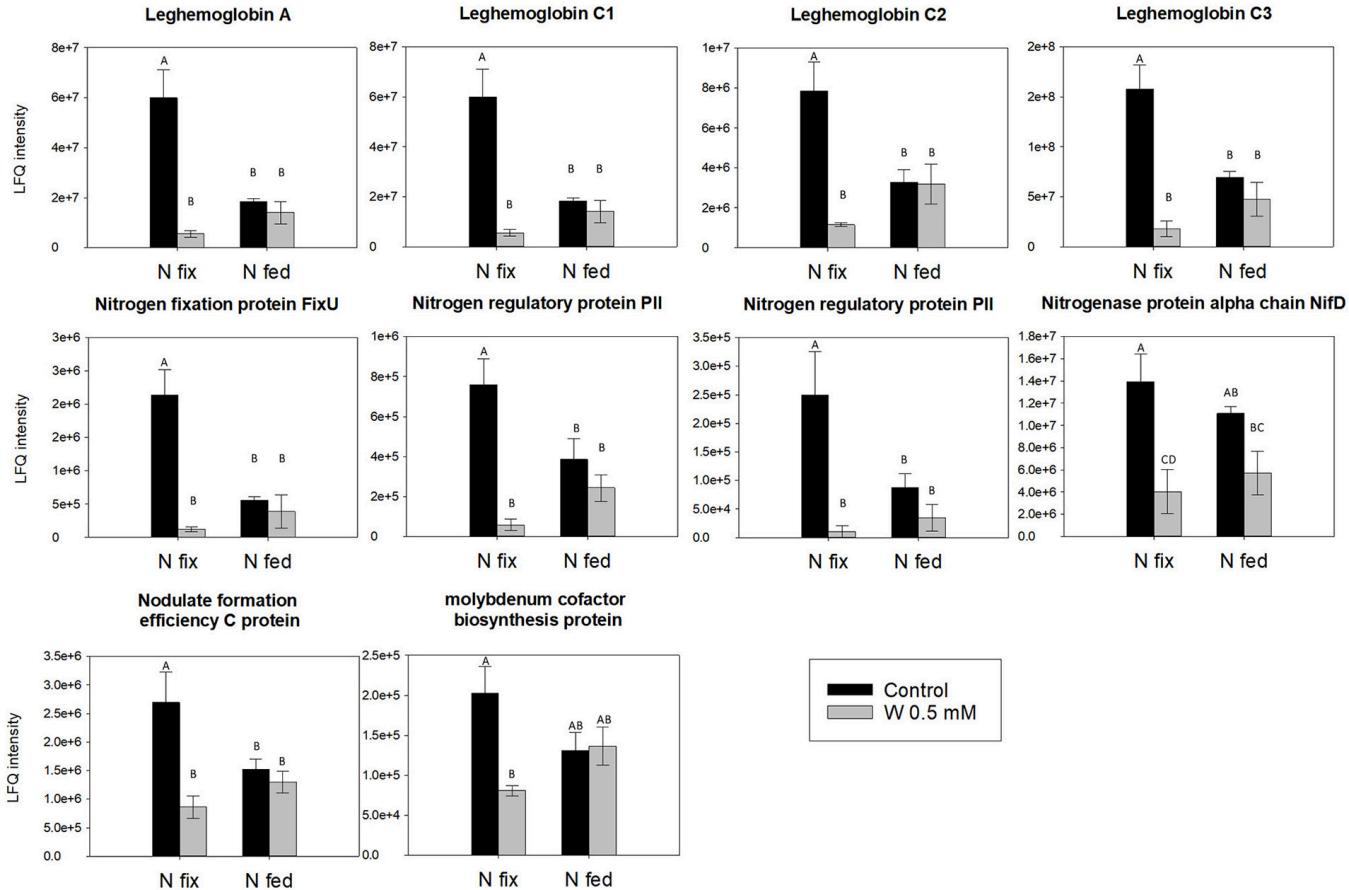
Relative abundance levels of N_2_-fixation relevant nodule proteins comparing two nitrogen regimes (N fix: week 2&3 0. 25 mM KNO_3_ & week 4–7 zero N; N fed: week 2–7 10 mM KNO_3_) and control and 0.5 mM W (W supplied as sodium tungstate). Letters indicate significant differences across the different W and N treatments (*ANOVA, post hoc Tukey, p* < 0.05). A detailed table with protein names and accession can be found in **Table S8C**.

The direct effects of W on the rhizobial nitrogenase, which also exhibits a Fe-Mo co-factor could not be tested with our approach. However, proteomics, data revealed that in N fix plants relative abundance levels of N_2_-fixation relevant proteins such as the nitrogenase precursors (NifD, NifT/FixU), a molybdenum cofactor biosynthesis protein (UniProt: G7D2E8) involved in the final step of Fe-Moco biosynthesis, as well as leghemoglobin, were strongly reduced in the presence of high W concentrations (~10-fold) (Figure 7 and Table S8C). NifD and leghemoglobin (Lb) are known to have an important role in abiotic stress response of nodules and have already been shown to be negatively affected by heavy metals such as Cd (Balestrasse et al., 2004; Marino et al., 2013). The decrease of the redox sensitive leghemoglobin potentially affects nitrogenase expression and thus activity by causing alterations in nodule oxygen concentrations (Marino et al., 2013). This is consistent with the depletion of proteins involved in Fe-Mo cofactor biosynthesis as well as the decline in the amount of N derived from N_2_ fixation in N fix plants and indicates that W might be indirectly affecting nitrogenase abundanceby inducing oxidative stress (Figures 2A, 7). Considering this decrease in abundance of proteins important for N_2_ fixation, the still considerable N_2_ fixation activity (mg N fixed g^−1^ nodule dwt.) suggests an increase in substrate turnover in W stressed plants. Furthermore, it is still not fully understood how Mo (and W) is taken up and transported into and between cells of higher plants as well as distributed or stored internally (Hagen, 2011; Bittner, 2014; Tejada-Jiménez et al., 2017; Vigani et al., 2017; Gil-Díez et al., 2018). Besides a proposed transport via sulfate transporters, three molybdate specific transporters MOT1(*Chlamydomas reinhartii and Arabidopsis thaliana*) and MOT2 (*Chlamydomas reinhartii*) have been identified (Tejada-Jiménez et al., 2013). More recently, two molybdenum transporters (MtMOT1.2 and a nodule-specific MtMOT1.3) have been identified in *Medicago truncatula*, living in symbiosis with *sinorhizobium meliloti*. While MtMOT1.2 is said to transport Mo through endodermis cells into the symplast, MtMOT1.3 was shown to mediate the transport into nodule cells and thus appears to be key in Mo supply for nitrogenase biosynthesis (Tejada-Jiménez et al., 2017; Gil-Díez et al., 2018). Interestingly, these two transporters are highly specific for molybdate anions, and appear not to be able to transport similar anions such as sulfate and possibly also tungstate (Gil-Díez et al., 2018). Furthermore, molybdenum an possibly also tungsten are proposed to be delivered to bacteroides via SST1 transporters and bacterial modABC transporters (Gil-Díez et al., 2018). Interestingly, we found rhizobial ABC transporters (mediating amino acid and phosphate transport), as well as proteins involved in ATP synthesis and N- and amino acid metabolism to be depleted at high tungsten (Table S7D).

Taken together, our data indicates that high W concentrations (0.5 mM) hamper N-assimilation and N_2_-fixation differently but to a similar degree with no clear advantage for either one of the N-regimes. However, our data suggests that while NR activity is inhibited by the incorporation of W into the enzyme, N_2_ fixation is indirectly affected by a decrease of Lb and NifD and thus reduced nitrogenase synthesis. This seems to be consistent with Nagatani and Brill (1974), who showed that although W reduced nitrogenase synthesis, specific activity of nitrogenase was only inhibited if Mo was omitted from the medium.

### Tungsten Stress Leads to Nutritional Imbalance

Similar to other HMs, the presence of increasing tungsten resulted in changes of nutrient tissue concentrations in soybean. Nutrient level changes induced by W could potentially be driven by (i) competition for uptake through the same transporters as other anions (phosphate, sulfate, molybdate), (ii) inhibition of uptake due to the reduction of root growth, (iii) decrease of nutrient availability due to polymerization with tungsten (particularly P and S) in soil solution and/or in acidic compartments of the cell such as the vacuole (Foy et al., 1978; Bednar et al., 2008; Johnson et al., 2010; Tran et al., 2010; Adamakis et al., 2012).

Several micronutrients (Mo, Cr, Fe, Cu) accumulated in the presence of high tungsten concentrations (0.5 mM W), particularly in soybean shoot tissue; however, these trends were not consistent across the different W and N treatments (Table 1). An increased micronutrient demand could be explained by their importance for molybdenum-specific metabolic processes, since there is a proposed crosstalk between molybdenum, copper and iron metabolism (Hänsch and Mendel, 2009). Copper as well as iron are involved in MoCo biosynthesis since some of the molybdoenzymes require iron prosthetic groups, and copper is essential for the formation of MoCo intermediates (Hänsch and Mendel, 2009; Bittner, 2014). In order for the plant to keep the nitrogen metabolism running, the production of NR apo-enzymes and thus enhanced uptake of Fe, Cu and Mo is required. Consequently, the lack of active NR combined with enhanced Fe, Cu and Mo uptake indicates that NR function rather than NR biosynthesis itself is disturbed (Heimer et al., 1969; Deng et al., 1989; Xiong et al., 2012). A similar tungsten induced increase in Mo shoot, root and nodule concentrations was also observed when soybean was grown on limed soils spiked with increasing concentrations of metallic W (Oburger et al., 2018). This is in line with Strigul et al. (2009) who found that the presence of tungsten promotes hyperaccumulation of molybdenum and vice versa. Fe is among the most important nutrients for N_2_-fixation as it is also needed for synthesis of the Fe-S complex of the nitrogenase as well as for leghemoglobin, which is the reason why iron levels are constitutively higher in the roots of N fix control plants (Tang et al., 1992). While iron levels increased in shoots of both treatments at W 0.5 mM, there was no significant change Fe concentrations in roots at the highest tungsten concentration compared with the respective control (Table 1). This in combination with the significant reduction in S levels in roots indicates that, unlike nitrate reduction, N_2_ fixation was negatively affected by decreased co-factor biosynthesis, which also corresponds to our proteomic data. The increase in Fe and Cu could have led to disturbances of the oxidative balance, since both are redox active transition metals that take part in fenton and fenton-like reactions, which have highly reactive oxygen species as a product and thus have detrimental effects on the organism (Mithöfer et al., 2004; Tran et al., 2010).

At high W concentrations, we found a significant decrease in levels of Mg, Ca and Mn in shoots, and Mg, K and S in roots of both N-regimes (Table 1). Nevertheless, concentrations of all decreased macro-nutrients (K, Mg, Mn, Ca, S) were within the physiological ranges reported in literature (Marschner, 2012). The decrease in root S concentrations, as indicated by our proteomic data, might be due to the depletion of several ABC transporters and ATPases in response to tungsten in N fix nodules and roots of both treatments (Table S7D). Since the cellular uptake and transport of W is suspected to be mediated by transporters of the same family as oxyanions such as sulfate and phosphate, the observed disturbances in sulfate uptake were most likely a result of tungsten induced depletion or blockage of these transporters (Bevers et al., 2009). Considering that plants have been shown to increase S uptake for increased production of phytochelatins such as glutathione during HM exposure (e.g., Cd) (Nocito et al., 2006). The found depletion, especially in roots, thus indicates reduced chelation and reduced capacity to combat oxidative stress (Ben Ammar et al., 2008). Besides limitation in S, also a decrease in Mg can have consequence for the plants toxicity resistance, since Mg is a prerequisite for the functionality of the enzyme glutathione synthetase, which is the key enzyme of glutathione biosynthesis, and has been shown to alleviate HM stress by decreasing negative electrical potential (Marschner, 2012; Rengel et al., 2015). Furthermore, Mg is most notably required as central atom of chlorophyll, as well as for a variety of different enzymes such as fructose-1.6-biphosphate, involved in sugar-starch partitioning and glutamine synthetase, involved in ammonia assimilation, as well as other physiologically important reactions such as transfer of phosphate and carboxyl groups (Marschner, 2012). Since Mn serves as co-factor in allantoate amidohydrase, a ureide degrading enzyme (allantoin, allantoate as major transport form of symbiotically fixed nitrogen in soy), the difference Mn leaf tissue concentrations between N fix and N fed can be explained by the higher demand for Mn of N_2_ fixing legumes (Marschner, 2012). Accordingly, The decrease of Mn in N fix leaf tissue at the highest W concentration might be due to the observed reduction of N_2_ fixation with increasing tissue W. Calcium, which was also decreased at high W, has important function as structural and regulatory component of macromolecules (especially cell walls), as well as in osmo-regulation and cation-anion balance and secondary messenger (Marschner, 2012). The cations Mg, Mn, K, and Ca are taken up by other channels and transporters than S and P (and prospectively also W), which indicates that W causes a depletion of these nutrients through other mechanisms. We could identify a potassium channel (UniRef100 = I1NEH6) which was significantly depleted at high W, which could explain the observed decrease in root K concentrations.

Although not reflected in total P concentrations, one reason for the decrease in macronutrient tissue concentrations (Mg, K, Ca, Mn) could be a W induced phosphate deficiency (Kavka and Polle, 2017). It is known that W polymerizes with itself or other ions in acidic environments (Strigul et al., 2010; Oburger et al., 2018) and also in biological systems (Johnson et al., 2010). Polymerization of W with phosphorous in the acidic compartments of the cell, such as the vacuole, which is where HMs but also nutrients are normally sequestered and stored, could lead to P immobilization and thus P deficiency. This would also explain the increase of P in roots in both N treatments as well as the accumulation of purple acid phosphatases in N fix nodules of the high tungsten treatments (Tran et al., 2010) (Table 1, Figure 4B). Acid phosphatases are enzymes that mediate vacuolar P cycling but also catalyze the hydrolysis of organically bound phosphate in extracellular environments and have been shown to increase under phosphate malnutrition as well as oxidative and salt stress (Tabaldi et al., 2007; Tran et al., 2010; Solanki and Dhankhar, 2011; Kavka and Polle, 2017). N_2_ fixing legumes possess higher acidic phosphatase activity under P limitation or at high N supply, as nodules are strong P sinks, and P is essential for both, nodulation and N_2_ fixation (Zahran, 1999; Png et al., 2017). Additionally, it has been shown that N_2_ fixation dependent plants require more phosphorus than those additionally supplied with nitrogen, which becomes especially critical for the symbionts during P limitation (Zahran, 1999). The fact that acid phosphatases only increased in N fix plants mirrors the higher demand of P for N fixation and indicates that the plants tried to meet the bacterial P requirement. This in turn potentially led to the observed maintenance of N_2_ fixation and thus the only slightly reduced N_2_ fixation activity.

### Tungsten Stress Provokes Carbon Immobilization

High W concentrations strongly increased starch content in shoots of both N regimes and in roots of N fed plant (Figure 3). It has already been reported that HM can lead to an accumulation of starch and soluble sugars accumulate in aerial plant parts due to HM induced blockage or/and reduction of source to sink transport (Pahlsson, 1989).

One reason for an increase in starch tissue concentration could be the aforementioned polymerization of tungsten and phosphate in the vacuole, since an often reported effect of phosphate starvation is an increase in starch and sucrose levels (Hermans et al., 2006; Rouached et al., 2010). Phosphate deficiency has direct effects on plant physiological processes such as glycolysis, respiration and photosynthesis, ATP-synthesis and calvin cycle, which were found to be significantly affected in our study (Figure 4B and Tables S4, S5, S7A,D). As discussed above, we additionally found an increase in root phosphorous and iron content as well as an increased root:shoot biomass ratio in N fix, all consistent with symptoms of phosphate starvation (Table 1 and Table S1) (Hammond and White, 2008; Rouached et al., 2010). Root biomass, nutrient and starch content data (Figures 1B, 3B, Table 1)indicates that N fix plants were better able to cope with W induced P starvation due to elevated levels of acidic phosphatases, which might have resulted in a maintained carbon supply to roots of N fix plants (Figure 1B).

Besides polymerization with nutrients (e.g., P), tungsten could bind to -SH groups of enzymes and other proteins (Sharma and Dietz, 2009; Aloui et al., 2011; Solanki and Dhankhar, 2011; Hossain et al., 2012), similar to other heavy metals. We found higher levels of alpha-amylase/subtilisin inhibitors (serine protease) and kunitz-trypsin protease inhibitory proteins as well as some respective proteases (subtilisin proteases) in the presence of high W in both N regimes, however, increase was more pronounced in N fed plants (Figure 6, Table S7C). The increase of these bifunctional protease inhibitory proteins could be another reason for the increased starch content. It has already been shown that during germination proteins involved in starch breakdown such as α-amylase, β-amylases are inhibited by heavy metals such as Zn, As, Cr and Cd (Rodríguez-Celma et al., 2010; Solanki and Dhankhar, 2011; DalCorso et al., 2013).

### Tungsten Induced Stress Responses

HMs have been reported to act as an impediment to normal metabolic functioning on various levels (Emamverdian et al., 2015). Several HM induced plant stress response mechanisms are not specific for HM stress but can be observed due to a variety of biotic and abiotic environmental stresses (e.g., herbivore and pathogen attack, salt, drought and logging).

We identified a set of twelve proteins that seem to be majorly involved in tungsten stress response, and accumulated in roots and nodules at high W, irrespective of N regime (Table 2). Most of them were general stress responsive proteins, so called pathogenesis related proteins (PR proteins), which have been shown to respond to pathogen attack and biotic stress, and to accumulate during abiotic stress such as nutrient deficiency or heavy metal stress (Didierjean et al., 1996; Maksymiec, 2007; Ahsan et al., 2009; Hossain and Komatsu, 2012; Sharmin et al., 2012). Among the identified proteins were two general PR proteins (C6T3A2, C6SYB8), a germin-like protein (C7S8D4), stress associated message SAM22 (C6T3A2) as well as a thaumatin-like protein (I1K6M2, I1L927). Thaumatin-like proteins are PR-5 group proteins that have been reported to be activated due to biotic as well as abiotic stress such as chemical treatment, high UV light, wounding, salt stress and HM toxicity (Frendo et al., 1992; Didierjean et al., 1996; Führs et al., 2008; Aloui et al., 2009; Tachi et al., 2009). The exact role of PR-5 group proteins is still unknown, it is proposed that they have similar functions to α-amylases/trypsin inhibitors, zeamatin (α-amylase inhibitors), osmotin-like proteins (increased salt and drought tolerance, leads to increases of proline and quenches ROS) as well as anti-fungal proteins (van Loon, 1985; Malehorn et al., 1994; Zhu et al., 1995; Koiwa et al., 1999; Kishor et al., 2015). Stress induced protein SAM22 (starvation-associated messessager) was found to be increased by various elicitors such as salicylic acid, hydrogen peroxide, methyl viologen or chitosan as well as depletion of cytokinin and auxin (Crowell et al., 1992). Germin-like proteins (GLPs), have been found to be accumulating due to pathogen attack, temperature stress, water stress, heavy metals and nutrient starvation (N, P, and K) but also during germination and senescence (Dunwell et al., 2008). Their exact function has so far not been described, however, they are proposed to have Mn-Superoxide dismutase (SOD) activity. SODs dismutate superoxide to H_2_O_2_ in order detoxify the reactive oxygen, an thus are proposed to be involved in cell wall stiffening by supplying H_2_O_2_ for lignification (Bernier and Berna, 2001; Dunwell et al., 2008). Accordingly, we found an increase of peroxidases (I1J7M4, Q9XFI8) and polyphenol oxidase (K7KJM5) across all treatments, and laccase (in roots) which mediate the formation of lignin from its precursors. Peroxidases and polyphenol oxidases are furthermore involved in detoxification of oxygen radicals (Pourcel et al., 2007). It is proposed that lignin biosynthesis is induced under HM stress in order to strengthen and stiffen cell walls and to reduce root growth, reducing uptake of the HM into the plant (Gall et al., 2015). Quite fittingly, we also observed that chitinases were increased in roots of both treatments. These, fungal and bacterial cell wall degenerating enzymes, are thought to be induced during HM stress by changes in cellular oxidative status (Javed et al., 2013) as they contribute to water retention, decrease cell wall elasticity and permeability for metals (Kasprzewska, 2003; Mészáros et al., 2014). However, in addition, we found another cell wall associated enzyme, a ß-expansin (I1MV71) to be highly responsive to tungsten. Interestingly, expansins lead to a loosening of the cell wall and expansion and thus cell growth, which seems antagonistic to the observed indices of lignification. Additionally, we observed increased levels of pectin esterases and annexin in nodules of N fix plant, which both have been shown to be involved in Al tolerance in *medicago truncatula* (Chandran et al., 2008). Pectin plays an essential role in HM binding and chelation. Pectin esterases mediate the de-esterification of pectin, resulting in higher affinity to bind to HM, and thus sequestering them into the cell wall (Gall et al., 2015). However, expansins have been reported to be involved in environmental stress tolerance (heat, drought, and nutrient stress i.e., increase in P uptake efficiency in soy) as well as to interact with cell wall bound peroxidases and thus to improve tolerance to oxidative stress (Marowa et al., 2016). This does not only hint at a tungsten induced oxidative imbalance but again, at an effect of tungsten stress on phosphate uptake and utilization which is also indicated by the increase of expansins (but also GLPs) since they have been shown to be involved in alleviation during phosphate deprivation. As discussed above, polymerization of P with W in the vacuole could lead to cellular phosphate depletion and thus an activation of the plants phosphate deficiency response. An expansin mediated increased P uptake efficiency would also explain the increase in phosphate concentrations in roots.

Since most of the above discussed proteins serve a variety of different functions, it cannot conclusively be answered which specific function they serve during W stress. It appears, however, that one major site of W stress response is the reorganization of the cell walls in roots. The accumulation of expansin concurrent with lignification of secondary cell walls could hint at two different strategies to alleviate W toxicity: I.) by chelation with pectin and sequestration into cell walls in nodules, possibly to reduce effects on N_2_ fixation, II.) by enforcing cell walls via lignification, making them less permeable for W (roots and nodules).

However, reactive oxygen species such as hydrogen peroxide (H_2_O_2_) and O${}_{2}^{-}$ are not only essential for lignification processes, they can also be transformed into highly reactive hydroxyl radicals in so called Fenton or Fenton-like reactions although in presence of redox active transition metals such as Cu(I), Fe(II) (Mithöfer et al., 2004), but also Cr III and VI (Sharmin et al., 2012) and, *in vitro*, also MoO${}_{4}^{2-}$ (Aubry and Cazin, 1988; Aubry et al., 1989; Boehme and Brauer, 1992; Popivker et al., 2013). The observed increase in abundance of a variety of peroxidases but also other enzymes involved in antioxidative defense (glutathione S-transferases (GST) in both N regimes; monodehydroascorbate reductase (MDHAR) in N fed plants) as well as an altered regulation of the glyoxalase system (lactoglutathione lyase in nodules of N fix only), (Tables S4, S5) indeed indicate that tungsten interferes with oxidative homeostasis by inducing accumulation of ROS. Interestingly, many of the above discussed PR proteins have been shown to also accumulate during oxidative stress (SAM22, Thaumatin-like, chitinases).

Excessive amounts of heavy metal ions have been found to lead to oxidative damage through (i) increased production of ROS, (ii) the aforementioned transformation of ROS (H_2_O_2_ and O${}_{2}^{-}$) to highly toxic hydroxyl radicals, (iii) protein degradation due to binding to –SH groups, (iv) inhibition of enzymatic activity, and (v) displacement of nutrient cations such as K, Ca, Mg, Fe and Mn from binding sites (Hall, 2002; Maksymiec, 2007; Ahsan et al., 2009; Hänsch and Mendel, 2009; Hossain and Komatsu, 2012). Since tungsten and molybdenum share certain chemical and structural similarities (Lassner et al., 2000; Koutsospyros et al., 2006; Bevers et al., 2009), excessive W might also contribute to ROS formation in a similar manner to Mo and other transition metals and take part in Fenton-like reactions. However, direct evidence for these reactions in presence MoO${}_{4}^{2-}$ to also happen in cellular environments, as well as between tungsten and H_2_O_2_ has not been presented to date.

Nonetheless, we also found that many proteins reported to be accumulating during oxidative stress such as superoxide dismutase (SOD), catalase, glutathione reductase, ascorbate peroxidase and thioredoxin remained unchanged or were depleted in both N regimes at the highest concentrations of tungsten (Tables S4, S5). Depending on HM-dose, growth conditions and organism, differential expression of SODs, APX, peroxidases and other enzymes involved in antioxidative defense was reported for several heavy metals, such as Cr (Sharmin et al., 2012), Cu (Lingua et al., 2012), Cd (Sandalio et al., 2001; Schützendübel et al., 2001; Ferreira et al., 2002; Sobkowiak and Deckert, 2006; Kieffer et al., 2009), As (Requejo and Tena, 2005) and Al (Guo et al., 2004; Sharma and Dietz, 2009). The depletion of antioxidative enzyme levels in our experiment (Tables S4, S5) might be due to the severity of stress induced by high (0.5 mM) W concentrations or the consumption of metabolites such as antioxidative glutathione and ascorbate via other paths (Sharmin et al., 2012). Accordingly, our results indicate that the detoxification of ROS via SODs and the glutathione-ascorbate cycle as well as catalase and peroxidases (Gratão et al., 2005; Hossain et al., 2012) was either blocked or dysfunctional at the administered tungsten concentration.

Furthermore, we observed an accumulation of glutathione S-transferases (GSTs) under W stress (Figure 4B), which were found to be increased in the presence of other HM like Al, AS, Cd and Cu (Marrs, 1996; Ahsan et al., 2009; Hossain et al., 2012). GSTs are said to have an antitoxic function by catalyzing the conjugation of GSH to electrophilic xenobiotics as well as endogenous secondary metabolites (e.g., anthocyanins, phytoalexins) for sequestration into the vacuole (Edwards et al., 2000; Pompella et al., 2003). In addition, they act as non-enzymatic carrier proteins for intracellular transport (e.g., IAA) and can possess a glutathione peroxidase activity and directly detoxify hydroxyl radicals (Marrs, 1996).

In order to combat oxidative stress, plants need to reduce ROS production by keeping the mitochondrial electron transport chain, a major source of ROS, sufficiently oxidized and bringing the system back into balance (Møller, 2001). The upregulation of glycolysis and TCA-cycle is a prerequisite for adequate NADH supply to the mitochondrial electron transport and can thus be expected (Kieffer et al., 2009; Hossain and Komatsu, 2012; Sharmin et al., 2012). However, our proteomic analysis revealed quite the opposite. Almost all key enzymes of glycolysis and TCA-cycle were depleted roots and nodules (Figure 4). This general metabolic breakdown apparently resulted in a disruption of the mitochondrial electron transport chain and ATP synthesis probably making it even more difficult for the cell to reinstate normal redox conditions. With minor exceptions, proteomic data from both N regimes showed this breakdown of TCA-cycle, glycolysis, lipid metabolism and mitochondrial electron transport (Figure 4B). Nevertheless, different sets of enzymes were depleted at 0.5 mM W depending on the mode of N supply. While in N fix and N fed roots of the high W treatment almost all key enzymes of primary metabolic pathways were depleted, there was an accumulation of some enzymes involved in glycolysis, lipid metabolism, TCA-cycle and amino acid metabolism in N fix nodules (Figure 4). This indicates that although metabolic processes of N fix and N fed plants were similarly affected, certain energy producing metabolic functions such as sugar metabolism were maintained or even enhanced in N fix nodules at high W concentrations, which might have contributed to uphold low levels of N_2_ fixation and plant growth (Figures 1,2).

### Symbiosis Specific Response

Our data indicated a symbiotically enhanced activity of hormone biosynthesis and secondary metabolism in nodules, not observed in roots in the presence of high W. In contrast to roots of N fed plants, where oxygen radicals were most likely scavenged by phenol/ascorbate/peroxide system, plants depending on functional symbiosis additionally enhanced flavonoid biosynthesis (chalcone synthase, chalcone isomerase, dihydroflavonol 4-reductase and isoflavone reductase), which also have been shown to function as radical scavengers (Table S5). This suggests different coping strategies between the two nitrogen treatments. Hale et al. (2002) showed that anthocyanins change color because of a complexation with either molybdenum or tungsten in *Brassica* and that they are most likely involved in the sequestration of transition elements to peripheral cell layers. However, the authors did not find a direct correlation between tungsten tolerance and anthocyanin content. In corn, flavonoids have been found to be involved in aluminum resistance and are thought to be able to chelate HM (Winkel-Shirley, 2002).

In nodules of the N fix treatment, lipoxygenases and 12-oxophytodienoate reductase, both enzymes involved in the biosynthesis of jasmonate (i.e., phytohormon) were accumulated. The jasmonate biosynthetic pathway has been found to be induced by a variety of biotic and abiotic stressors such as wounding, osmotic stress, herbivore attack as well as heavy metals (Maksymiec, 2007). Noteworthy, they were recently observed to be induced in leaves of N fix compared to non-nodulated N-fertilized *Medicago truncatula* plants and proposed to be involved in drought stress alleviation (Staudinger et al., 2016). An increase in jasmonic acid and methyl jasmonate results in a decrease in growth and photosynthetic activity, as well as an increased production of secondary metabolites (e.g., flavonols) and an accumulation of so called jasmonate inducible proteins (JiPs) (Maksymiec et al., 2005). Such Jips, two Kunitz-trypsin protease inhibitors (I1KW54, I1MI59), were among the 12 identified tungsten responsive proteins that were accumulating across all organs and treatments. Alpha-amylase/subtilisin inhibitor activity was reported to be induced by various heavy metals as well as other abiotic stressors (Frendo et al., 1992; Solanki and Dhankhar, 2011). In drought tolerant soybean Kunitz trypsin protease inhibitors and acid phosphatase showed a significant increase (37-fold and 114-fold increase respectively) during severe drought (Yu et al., 2016). Our data suggests that both proteins are also involved in tungsten stress response.

Even though, severe W stress seems to affect *G. max* independent of symbiotic functioning, these findings suggest a possible explanation as to how N fix plants maintained their root growth and nodule fixation activity at lower W levels.

## Conclusion

Our study confirmed the negative impact of W on N-assimilation via nitrate reduction. Furthermore, we demonstrated that N_2_ fixation was significantly impaired by reduced nodulation and an overall reduced nitrogenase abundance (NifD) at high W. Although, we cannot fully exclude a direct inhibition of nitrogenase activity by substitution of Mo by W as co-factor, our data indicates that W affects nitrogenase rather indirectly by reduced enzyme synthesis due to altered nodule oxygen concentrations. In order to conclusively answer this question, and to better understand transport and cellular fate of tungsten, further experiments, using imaging techniques such as NanoSims, are needed. Thus, our study showed that—when exposed to high W concentrations—functional symbiotic association with *Bradyrhizobium japonicum* does not result in an increased resistance to W compared to nitrate fed soy bean plants with reduced symbiotic interaction.

*G. max* was able to take up considerable amounts of W (703 ± 136 mg kg^−1^) when exposed to high W concentrations (0.5 mM). Even though, our data suggest that functional symbiotic association with *Bradyrhizobium japonicum* does not result in an increased resistance to high W, there were indices for a potential tolerance mechanisms at lower W levels, such as a symbiont mediated increase in secondary metabolic processes and hormone biosynthesis.

Our study provides evidence that metabolic processes aside from molybdoenzymes are targeted by W toxicity. We observed that tungsten induced the immobilization of sugar storage pools, lead to significant alterations in levels, and distribution of major plant nutrients (S, Mg, Mn, P, Ca, K, Mo) as well as a growth inhibition. Furthermore, W toxicity caused the induction of several common stress response mechanisms headed by the accumulation of peroxidases and protease inhibitors. Having identified some robust key targets of tungsten toxicity in soy bean, by stringent filtering and validation, a future goal might be to identify and clarify their potential function in tungsten stress regulation.

## Data Availability

The mass spectrometry proteomics data have been deposited to the ProteomeXchange Consortium via the PRIDE (Vizcaíno et al., 2016) partner repository (https://www.ebi.ac.uk/pride/archive/) with the dataset identifier PXD010877.

## Author Contributions

JP performed experiments, data analysis and took the lead in writing the paper. SW was involved in planning and supervised the work and wrote the paper. EO conceived the study, was in charge of overall direction and planning, performed data analysis and wrote the paper. WW wrote the paper. All authors provided critical feedback and helped shape the research, read and approved the paper.

### Conflict of Interest Statement

The authors declare that the research was conducted in the absence of any commercial or financial relationships that could be construed as a potential conflict of interest.
