# Supplementary material for: Molecular Mechanisms of Tungsten Toxicity Differ for Glycine max Depending on Nitrogen Regime
Source: Front Plant Sci. 2019 Apr 2;10:367. doi: 10.3389/fpls.2019.00367 (PMC6454624; doi:10.3389/fpls.2019.00367)
Supplement: Supplementary file 1 [file Table_1.docx]

Supplementary Material

Molecular mechanisms of tungsten toxicity differ for *Glycine max* depending on mode of nitrogen regimes

Julian Preiner^1,2^, Stefanie Wienkoop^1*^, Wolfram Weckwerth^1^, Eva Oburger^2,3^

^1^ Devision of Molecular Systems Biology, Department of Ecogenomics and Systems Biology, University of Vienna, Vienna, Austria

^2^ Institute of Soil Research, Department of Forest and Soil Sciences, University of Natural Resources and Life Sciences Vienna, Tulln, Austria

3 Devision of Terrestrial Ecosystem Research, Department of Microbiology and Ecosystem Science, University of Vienna, Vienna, Austria

*** Correspondence:**Stefanie Wienkoop
stefanie.wienkoop@univie.ac.at

# Supplementary Tables

**SI Table 1** A) R:S ratio B) W TF factor of soybean (*Glycine max* cv Primus) grown semi-hydroponically with increasing W concentrations (control, 0.1, 0.5 mM W supplied as sodium tungstate) and under differing nitrogen supply regimes (N fix: week 2&3 0. 25 mM KNO_3_ & week 4-7 zero N; N fed: week 2-7 10 mM KNO_3_). Letters indicate significant differences across the different W and N treatments (*ANOVA, Post Hoc DGC*, *p*<0.05). LOQ – limit of quantification.

| **R:S** | **N fix** | | | | **N fix** | | | | **N fix** | | | | **N fed** | | | | **N fed** | | | | **N fed** | | | |
| --- | --- | --- | --- | --- | --- | --- | --- | --- | --- | --- | --- | --- | --- | --- | --- | --- | --- | --- | --- | --- | --- | --- | --- | --- |
|  | **Control** | | | | **0.1 mM W** | | | | **0.5 mM W** | | | | **Control** | | | | **0.1 mM W** | | | | **0.5 mM W** | | | |
| ratio | 0.15 | ± | 0.02 | **C** | 0.28 | ± | 0.02 | **B** | 0.45 | ± | 0.04 | **A** | 0.22 | ± | 0.01 | **B** | 0.23 | ± | 0.02 | **A** | 0.27 | ± | 0.05 | **A** |
| **TF** | **N fix** | | | | **N fix** | | | | **N fix** | | | | **N fed** | | | | **N fed** | | | | **N fed** | | | |
|  | **Control** | | | | **0.1 mM W** | | | | **0.5 mM W** | | | | **Control** | | | | **0.1 mM W** | | | | **0.5 mM W** | | | |
| W | <LOQ | | | **C** | 0.66 | ± | 0.06 | **B** | 0.58 | ± | 0.12 | **B** | <LOQ | | | **C** | 1.02 | ± | 0.06 | **A** | 0.87 | ± | 0.3 | **A** |

**SI Table 2** nodule concentrations of tungsten, as well as macro- and micronutrients of soybean (*Glycine max* cv Primus) grown semi-hydroponically with increasing W concentrations (control, 0.1, 0.5 mM W supplied as sodium tungstate) and under differing nitrogen supply regimes (N fix: week 2&3 0. 25 mM KNO_3_ & week 4-7 zero N; N fed: week 2-7 10 mM KNO_3_). LOQ – limit of quantification, n.d. for “not determined due to insufficient biomass”, n.r. for “not determined due to number of replicates”.

| **Nodules** | **unit** | **N fix** | | | | **N fix** | | | | **N fix** | | | | **N fed** | | | | **N fed** | | | | **N fed** | | | |
| --- | --- | --- | --- | --- | --- | --- | --- | --- | --- | --- | --- | --- | --- | --- | --- | --- | --- | --- | --- | --- | --- | --- | --- | --- | --- |
|  |  | **Control** | | | | **0.1 mM W** | | | | **0.5 mM W** | | | | **Control** | | | | **0.1 mM W** | | | | **0.5 mM W** | | | |
|  |  | **n=6** | | | | **n=4** | | | | **n=1** | | | | **n=1** | | | | **n=1** | | | | **n=0** | | | |
| **W** | mg kg^-1^ | <LOQ | | |  | 895.21 | ± | 50.94 |  | 1248.65 | ± | n.r. |  | <LOQ | | |  | 677.19 | ± | n.r. |  | n.d. | ± | n.d. |  |
| **Mo** | mg kg^-1^ | 12.01 | ± | 0.94 |  | 7.44 | ± | 1.15 |  | 2.28 | ± | n.r. |  | 6.15 | ± | n.r. |  | 7.66 | ± | n.r. |  | n.d. | ± | n.d. |  |
| **Cu** | mg kg^-1^ | 15.53 | ± | 1.15 |  | 14.92 | ± | 1.41 |  | 14.33 | ± | n.r. |  | 21.15 | ± | n.r. |  | 13.46 | ± | n.r. |  | n.d. | ± | n.d. |  |
| **Cr** | mg kg^-1^ | 5.49 | ± | 0.94 |  | 5.43 | ± | 1.15 |  | 3.96 | ± | n.r. |  | 8.81 | ± | n.r. |  | 9.90 | ± | n.r. |  | n.d. | ± | n.d. |  |
| **Mn** | mg kg^-1^ | 16.76 | ± | 1.90 |  | 14.64 | ± | 2.32 |  | 10.02 | ± | n.r. |  | 27.17 | ± | n.r. |  | 16.25 | ± | n.r. |  | n.d. | ± | n.d. |  |
| **Fe** | g kg^-1^ | 0.64 | ± | 0.03 |  | 0.44 | ± | 0.04 |  | 0.35 | ± | n.r. |  | 0.66 | ± | n.r. |  | 43.50 | ± | n.r. |  | n.d. | ± | n.d. |  |
| **P** | g kg^-1^ | 4.73 | ± | 0.21 |  | 4.04 | ± | 0.26 |  | 2.81 | ± | n.r. |  | 2.39 | ± | n.r. |  | 2.47 | ± | n.r. |  | n.d. | ± | n.d. |  |
| **Mg** | g kg^-1^ | 8.18 | ± | 0.56 |  | 4.81 | ± | 0.69 |  | 1.82 | ± | n.r. |  | 5.38 | ± | n.r. |  | 2.61 | ± | n.r. |  | n.d. | ± | n.d. |  |
| **S** | g kg^-1^ | 7.93 | ± | 0.25 |  | 4.88 | ± | 0.30 |  | 2.13 | ± | n.r. |  | 7.17 | ± | n.r. |  | 3.93 | ± | n.r. |  | n.d. | ± | n.d. |  |
| **Ca** | g kg^-1^ | 0.79 | ± | 0.24 |  | 0.45 | ± | 0.29 |  | 0.36 | ± | n.r. |  | 0.42 | ± | n.r. |  | 0.11 | ± | n.r. |  | n.d. | ± | n.d. |  |
| **K** | g kg^-1^ | 13.48 | ± | 0.83 |  | 13.18 | ± | 1.01 |  | 9.49 | ± | n.r. |  | 18.00 | ± | n.r. |  | 13.15 | ± | n.r. |  | n.d. | ± | n.d. |  |

**SI Table 3** List of significantly changed proteins in both organs (roots, nodules) and N regimes (N fix: week 2&3 0. 25 mM KNO_3_ & week 4-7 zero N; N fed: week 2-7 10 mM KNO_3_) between control and high tungsten (0.5 mM W, supplied as sodium tungstate). Asterisk indicate significant difference (*ANOVA, Post Hoc Tukey*, *p*<0.05). Proteins uniquely identified by prototypic peptides are indicated with an x.

| **Table 6. Proteins, significant in all organs and all treatments** | | | | | **N fix** | | | | | |  | **N fed** | | | | | |
| --- | --- | --- | --- | --- | --- | --- | --- | --- | --- | --- | --- | --- | --- | --- | --- | --- | --- |
| **ORGAN** | **Uniprot ID** | **proteotypic evaluation** | **Mercator categories** | **Annotation (UniprotKB/Uniref100)** | **ztrans LFQ int.** | | | | | |  | **ztrans LFQ int.** | | | | | |
|  |  |  |  |  | **C** | **W 500** | **ratio** | **p-value** | **BH adj. p-value** | **sig.** |  | **C** | **W 500** | **ratio** | **p-value** | **BH adj. p-value** | **sig.** |
| ROOT | **I1KQ26** |  | secondary metabolism | Uncharacterized Protein | 0.5 | -0.9 | 7.608 | 0.042 | 0.195 | * |  | 1.1 | -0.8 | 9.307 | 0.003 | 0.046 | ** |
| NODULE | **I1KQ26** |  | secondary metabolism | Uncharacterized Protein | -1.2 | 0.8 | 0.136 | 0.000 | 0.003 | *** |  | -0.4 | 0.9 | 0.489 | 0.001 | 0.177 | ** |
| ROOT | **I1KW53** |  | protease inhibitor | Uncharacterized Protein | -0.8 | 0.3 | 0.107 | 0.047 | 0.206 | * |  | -0.8 | 1.3 | 0.043 | 0.000 | 0.007 | *** |
| NODULE | **I1KW53** |  | protease inhibitor | Uncharacterized Protein | -0.8 | 1.1 | 0.312 | 0.001 | 0.017 | *** |  | -0.8 | 0.5 | 0.396 | 0.007 | 0.390 | ** |
| ROOT | **I1MI59** |  | protease inhibitor | kunitz- trypsin inhibitor | -0.9 | 0.9 | 0.017 | 0.000 | 0.005 | *** |  | -0.9 | 0.8 | 0.009 | 0.000 | 0.004 | *** |
| NODULE | **I1MI59** |  | protease inhibitor | kunitz- trypsin inhibitor | -0.8 | 1.3 | 0.002 | 0.000 | 0.00 | *** |  | -0.8 | 0.3 | 0.005 | 0.000 | 0.04 | *** |
| ROOT | **I1MV71** | **x** | cell wall | expansin-like B1 | -0.8 | 0.4 | 0.007 | 0.000 | 0.005 | *** |  | -0.8 | 1.2 | 0.003 | 0.000 | 0.000 | *** |
| NODULE | **I1MV71** | **x** | cell wall | expansin-like B1 | -0.8 | 1.3 | 0.028 | 0.000 | 0.00 | *** |  | -0.8 | 0.3 | 0.017 | 0.000 | 0.00 | *** |
| ROOT | **O48601** |  | secondary metabolism | NADPH:isoflavone reductase | 0.9 | -1.0 | 3.866 | 0.002 | 0.035 | ** |  | 0.9 | -0.7 | 2.781 | 0.007 | 0.068 | ** |
| NODULE | **O48601** |  | secondary metabolism | NADPH:isoflavone reductase | -0.9 | 0.8 | 0.008 | 0.028 | 0.159 | * |  | -0.8 | 0.9 | 0.064 | 0.017 | 0.605 | * |

**SI Table 4** List of significantly changed root proteins between control and high tungsten (0.5 mM W, supplied as sodium tungstate) of soybean (*Glycine max* cv Primus) grown semi-hydroponically under differing nitrogen supply regimes (N fix: week 2&3 0. 25 mM KNO_3_ & week 4-7 zero N; N fed: week 2-7 10 mM KNO_3_). Asterisk indicate significant difference (*ANOVA, Post Hoc Tukey*, *p*<0.05). Proteins uniquely identified by prototypic peptides are indicated with an x.

**SI Table 5** List of significantly changed nodule proteins between control and high tungsten (0.5 mM W, supplied as sodium tungstate) of soybean (*Glycine max* cv Primus) grown semi-hydroponically under differing nitrogen supply regimes (N fix: week 2&3 0. 25 mM KNO_3_ & week 4-7 zero N; N fed: week 2-7 10 mM KNO_3_). Asterisk indicate significant difference (*ANOVA, Post Hoc Tukey*, *p*<0.05). Proteins uniquely identified by prototypic peptides are indicated with an x.

**SI Table 6 (A)** List of nodule proteins above and below the cutoff for significantly changed root and nodule proteins that were responsible for PC1 and separate the control and high tungsten treatment (0.5 mM W, supplied as sodium tungstate) of soybean (*Glycine max* cv Primus) grown semi-hydroponically under differing nitrogen supply regimes (N fix: week 2&3 0. 25 mM KNO_3_ & week 4-7 zero N; N fed: week 2-7 10 mM KNO_3_). **(B)** List of root proteins above and below the cutoff for significantly changed root and nodule proteins that were responsible for PC1 and separate the control and high tungsten treatment (0.5 mM W, supplied as sodium tungstate) of soybean (*Glycine max* cv Primus) grown semi-hydroponically under differing nitrogen supply regimes (N fix: week 2&3 0. 25 mM KNO_3_ & week 4-7 zero N; N fed: week 2-7 10 mM KNO_3_). Asterisk indicate significant difference (*ANOVA, Post Hoc Tukey*, *p*<0.05). Proteins uniquely identified by prototypic peptides are indicated with an x.

**SI Table 7 (A)** List of significantly changed root and nodule proteins between control and high tungsten (0.5 mM W, supplied as sodium tungstate) of soybean (*Glycine max* cv Primus) grown semi-hydroponically under differing nitrogen supply regimes (N fix: week 2&3 0. 25 mM KNO_3_ & week 4-7 zero N; N fed: week 2-7 10 mM KNO_3_) assigned to the functional category “sugar metabolism”. Asterisk indicate significant difference (*ANOVA, Post Hoc Tukey*, *p*<0.05). Proteins uniquely identified by prototypic peptides are indicated with an x. **(B)** List of significantly changed nodule proteins between control and high tungsten (0.5 mM W, supplied as sodium tungstate) of soybean (*Glycine max* cv Primus) grown semi-hydroponically under differing nitrogen supply regimes (N fix: week 2&3 0. 25 mM KNO_3_ & week 4-7 zero N; N fed: week 2-7 10 mM KNO_3_) assigned to the functional category “RNA”. Asterisk indicate significant difference (*ANOVA, Post Hoc Tukey*, *p*<0.05). Proteins uniquely identified by prototypic peptides are indicated with an x. **(C)** List of significantly changed root and nodule proteins between control and high tungsten (0.5 mM W, supplied as sodium tungstate) of soybean (*Glycine max* cv Primus) grown semi-hydroponically under differing nitrogen supply regimes (N fix: week 2&3 0. 25 mM KNO_3_ & week 4-7 zero N; N fed: week 2-7 10 mM KNO_3_) assigned to the functional category “protease inhibitors”. Asterisk indicate significant difference (*ANOVA, Post Hoc Tukey*, *p*<0.05). Proteins uniquely identified by prototypic peptides are indicated with an x. **(D)** List of significantly changed root and nodule proteins between control and high tungsten (0.5 mM W, supplied as sodium tungstate) of soybean (*Glycine max* cv Primus) grown semi-hydroponically under differing nitrogen supply regimes (N fix: week 2&3 0. 25 mM KNO_3_ & week 4-7 zero N; N fed: week 2-7 10 mM KNO_3_) assigned to the functional category “transport and ABC transport”. Asterisk indicate significant difference (*ANOVA, Post Hoc Tukey*, *p*<0.05). Proteins uniquely identified by prototypic peptides are indicated with an x.

**SI Table 8 (A)** List of significantly changed root proteins between the different nitrogen supply regimes (N fix: week 2&3 0. 25 mM KNO_3_ & week 4-7 zero N; N fed: week 2-7 10 mM KNO_3_) of soybean (*Glycine max* cv Primus) grown semi-hydroponically under control conditions and high tungsten (0.5 mM W, supplied as sodium tungstate). Asterisk indicate significant difference (*ANOVA, Post Hoc Tukey*, *p*<0.05). Proteins uniquely identified by prototypic peptides are indicated with an x. **(B)** List of significantly changed nodule proteins between the different nitrogen supply regimes (N fix: week 2&3 0. 25 mM KNO_3_ & week 4-7 zero N; N fed: week 2-7 10 mM KNO_3_) of soybean (*Glycine max* cv Primus) grown semi-hydroponically under control conditions and high tungsten (0.5 mM W, supplied as sodium tungstate). Asterisk indicate significant difference (*ANOVA, Post Hoc Tukey*, *p*<0.05). Proteins uniquely identified by prototypic peptides are indicated with an x. **(C)** List of significantly changed N_2_-fixation relevant nodule and root proteins between the different nitrogen supply regimes (N fix: week 2&3 0. 25 mM KNO_3_ & week 4-7 zero N; N fed: week 2-7 10 mM KNO_3_) of soybean (*Glycine max* cv Primus) grown semi-hydroponically under control conditions and high tungsten (0.5 mM W, supplied as sodium tungstate). Asterisk indicate significant difference (*ANOVA, Post Hoc Tukey*, *p*<0.05). Proteins uniquely identified by prototypic peptides are indicated with an x.

# Supplementary Figures

**SI Figure 1 (A)** Pictures five week old soy bean plants comparing the two nitrogen regimes **1)** N fix and **2)** N fed (Nfix: week 2&3 0. 25 mM KNO_3_ & week 4-7 zero N; N fed: week 2-7 10 mM KNO_3_) and control, 0.1 mM W and 0.5 mM W (W supplied as sodium tungstate). **(B)** Pictures of roots comparing the two nitrogen regimes (N fix: week 2&3 0. 25 mM KNO_3_ & week 4-7 zero N; N fed: week 2-7 10 mM KNO_3_) and control, 0.1 mM W and 0.5 mM W (W supplied as sodium tungstate).

**SI Figure 2** Venn-diagram showing number of significantly changed (*ANOVA, Post Hoc Tukey*, *p*<0.05) root and nodule proteins comparing control with high W (0.5 mM W, supplied as sodium tungstate) of both nitrogen regimes (N fix: week 2&3 0. 25 mM KNO_3_ & week 4-7 zero N; N fed: week 2-7 10 mM KNO_3_).

**SI Figure 3** **(A)** Venn-diagram showing number of significantly changed (*ANOVA, Post Hoc Tukey*, *p*<0.05) root and nodule proteins comparing control with high W (0.5 mM W, supplied as sodium tungstate) of both nitrogen treatments. **(B)** Functional categories of 57 proteins that significantly changed in both organs. “Misc.” includes categories with only one protein changed each (Biodegradation of Xenobiotics, Cell, Gluco-, galacto- and mannosidases, Hormone metabolism, Lipid metabolism, Nucleotide metabolism, Oxidases - copper, flavone etc., Protein synthesis, Redox, S-assimilation, RNA).

**SI Figure 4 (A) Heatmap with clustering (Euclidean distance) of proteins with functional category stress.** (N fix: week 2&3 0. 25 mM KNO_3_ & week 4-7 zero N; N fed: week 2-7 10 mM KNO_3_) and control, 0.1 mM W and 0.5 mM W (W supplied as sodium tungstate). Cluster 6 and 5 show selectively accumulating clusters according to N regime. **(B) Protein groups above and below the chosen PC1** cutoff for significantly changed root and nodule proteins assigned to molecular function “stress” that were responsible for the variance (PC1, see Figure 5A–roots & Figure 5B-nodules) between the two tungsten treatments.

**
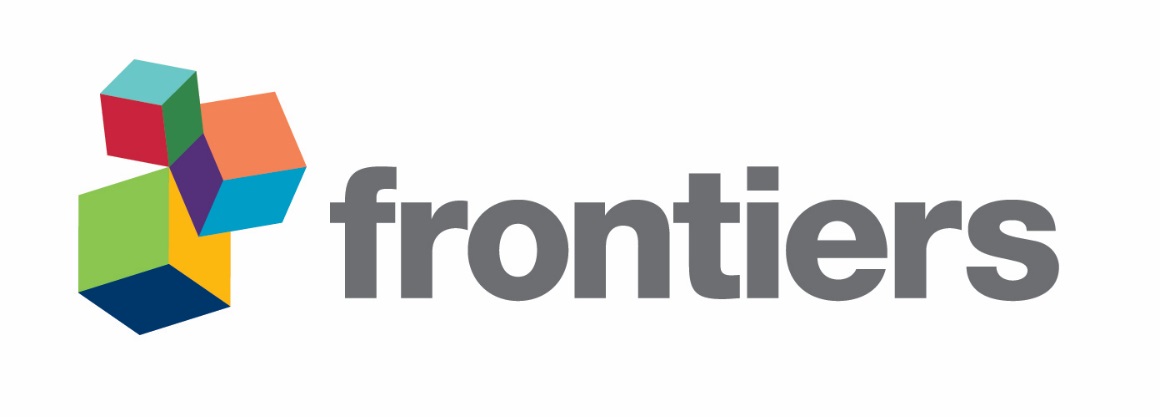
**
