# Supplementary figures and images for: Molecular Mechanisms of Tungsten Toxicity Differ for Glycine max Depending on Nitrogen Regime
Source: Front Plant Sci. 2019 Apr 2;10:367. doi: 10.3389/fpls.2019.00367 (PMC6454624; doi:10.3389/fpls.2019.00367)

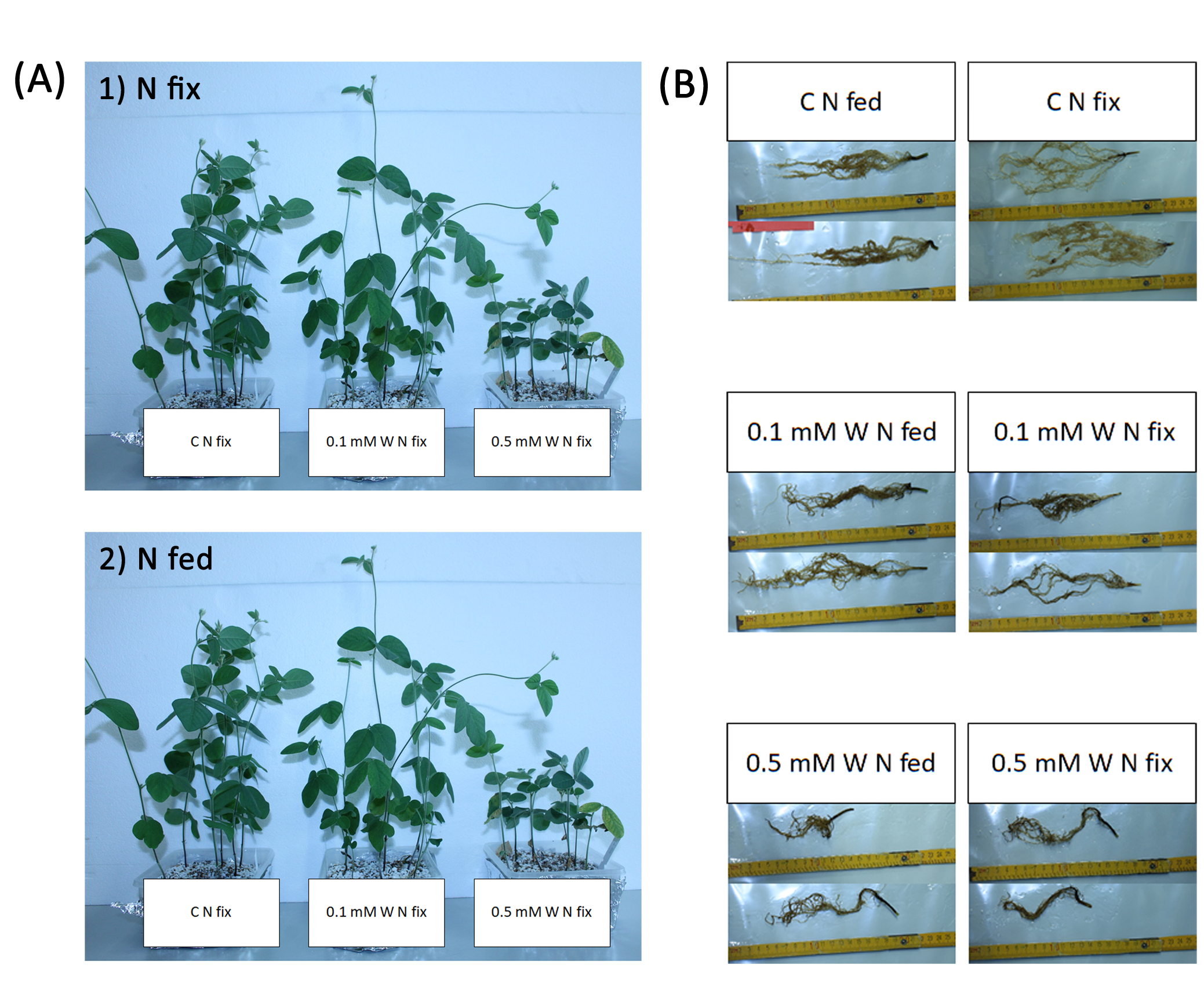

Supplement: Supplementary file 7 [file Image_1.TIF]

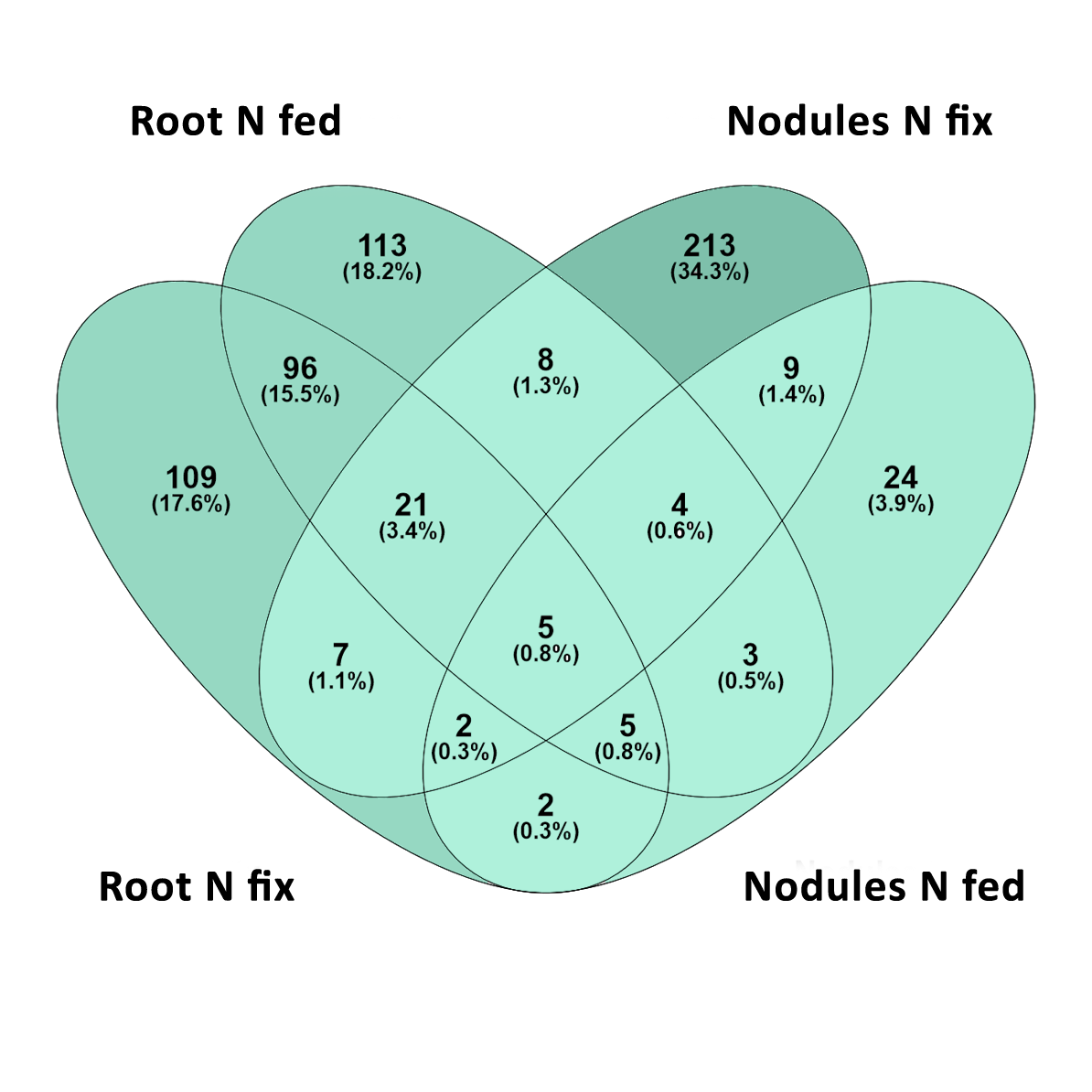

Supplement: Supplementary file 8 [file Image_2.TIF]
